# The Costs, Benefits, and Cost-Effectiveness of Interventions to Reduce Maternal Morbidity and Mortality in Mexico

**DOI:** 10.1371/journal.pone.0000750

**Published:** 2007-08-15

**Authors:** Delphine Hu, Stefano M. Bertozzi, Emmanuela Gakidou, Steve Sweet, Sue J. Goldie

**Affiliations:** 1 Program in Health Decision Science, Department of Health Policy and Management, Harvard School of Public Health, Boston, Massachusetts, United States of America; 2 Instituto Nacional de Salud Publica, Cuernavaca, Morelos, Mexico; 3 Harvard Initiative for Global Health, Cambridge, Massachusetts, United States of America; State University of New Jersey, United States of America

## Abstract

**Background:**

In Mexico, the lifetime risk of dying from maternal causes is 1 in 370 compared to 1 in 2,500 in the U.S. Although national efforts have been made to improve maternal services in the last decade, it is unclear if Millennium Development Goal 5 - to reduce maternal mortality by three-quarters by 2015 - will be met.

**Methodology/Principal Findings:**

We developed an empirically calibrated model that simulates the natural history of pregnancy and pregnancy-related complications in a cohort of 15-year-old women followed over their lifetime. After synthesizing national and sub-national trends in maternal mortality, the model was calibrated to current intervention-specific coverage levels and validated by comparing model-projected life expectancy, total fertility rate, crude birth rate and maternal mortality ratio with Mexico-specific data. Using both published and primary data, we assessed the comparative health and economic outcomes of alternative strategies to reduce maternal morbidity and mortality. A dual approach that increased coverage of family planning by 15%, and assured access to safe abortion for all women desiring elective termination of pregnancy, reduced mortality by 43% and was cost saving compared to current practice. The most effective strategy added a third component, enhanced access to comprehensive emergency obstetric care for at least 90% of women requiring referral. At a national level, this strategy reduced mortality by 75%, cost less than current practice, and had an incremental cost-effectiveness ratio of $300 per DALY relative to the next best strategy. Analyses conducted at the state level yielded similar results.

**Conclusions/Significance:**

Increasing the provision of family planning and assuring access to safe abortion are feasible, complementary and cost-effective strategies that would provide the greatest benefit within a short-time frame. Incremental improvements in access to high-quality intrapartum and emergency obstetric care will further reduce maternal deaths and disability.

## Introduction

Every year over half a million women die from complications of pregnancy and childbirth [1]. The maternal mortality ratio (MMR) for Mexico is estimated at 83 maternal deaths per 100,000 live births, nearly five times the ratio reported for the United States, and the lifetime risk of dying from maternal causes is 1 in 370 [1]. Within Mexico, there is substantial heterogeneity in maternal mortality, with the highest rates observed in Guerrero and the State of Mexico and the lowest rates in Colima and Sonora [2], [3].

Significant national efforts have been made to improve the coverage, quality, and range of maternal services for women in Mexico such as Fair Start in Life (Arranque Parejo en la Vida), the People's Health Insurance (Seguro Popular de Salud) and the Oportunidades (formerly *PROGRESA*) program [4]. To assist in programmatic and budgetary planning, the financial costs of providing the package of services included in the World Health Organization's (WHO) Mother Baby Package (MBP) at recommended coverage levels were estimated for Morelos state by the National Institute of Public Health [4], [5]. To date, however, few cost-effectiveness analyses of safe motherhood strategies in Mexico have been conducted from a societal perspective and long-term investment approach.

Information on the cost-effectiveness of alternative strategies to improve maternal care can serve as one important policy input to guide decisions on how to achieve Millennium Development Goal 5 (MDG5) of reducing the maternal mortality ratio by three-quarters between 1990 and 2015. Cost-effectiveness analyses can enhance and complement a national strategy to mount political commitment and evidence-based action. A modeling approach within a decision-analytic framework can combine information from a wide variety of sources, extrapolate costs and health effects beyond the time horizon of a single clinical study, and evaluate multiple potential interventions packaged into strategies. Using the best available clinical and epidemiologic data from Mexico and Latin America, we adopt this approach to conduct a cost-effectiveness analysis of alternative strategies to reduce maternal mortality and morbidity in Mexico.

## Methods

### Analytic Overview

We conducted a cost-effectiveness analysis of alternative maternal morbidity and mortality reduction strategies in Mexico using a computer-based model that simulates the natural history of pregnancy (both planned and unintended) and pregnancy-related complications in a cohort of 15-year-old women followed over their lifetime. Using regional and country-specific data, we compare the health outcomes and costs associated with the current maternal health intervention coverage levels (referred to as *current standard of care* or *current practice*) in Mexico; upgrading selected strategies to achieve coverage levels recommended in the WHO Mother Baby Package (*MBP*) standard of care; and increasing coverage of selected interventions alone (e.g., enhanced access to safe abortion) and in the context of strategic packages (e.g., increased family planning, enhanced safe abortion, improved access to emergency obstetric services). Model outcomes include intermediate clinical events (e.g., unsafe abortion, severe preeclampsia/eclampsia, obstructed labor, hemorrhage, and sepsis) and long-term outcomes (e.g., life expectancy, disability-adjusted life expectancy, and lifetime costs). We follow recommendations in published guidelines for standardizing economic evaluations [6]–[9]. The comparative performance of alternate strategies is described using the incremental cost-effectiveness ratio, defined as the additional cost of a specific strategy divided by its additional clinical benefit, compared with the next least expensive strategy. Sensitivity analyses assess the effect of varying baseline estimates and assumptions on our results.

### The Model

**Figure 1:**
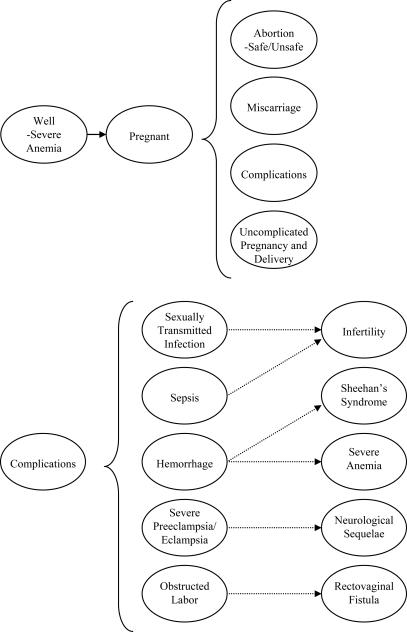
Schematic of Natural History Model. Upper Panel. The ovals represent the key health states used in the model. Nonpregnant 15-year-old women enter the model and are subject to an annual risk of pregnancy. Once pregnant, a woman may experience a miscarriage, elect to undergo an abortion, develop a maternal complication, or have an uncomplicated pregnancy and delivery. A small proportion of nonpregnant women will have severe anemia and subsequently will have a higher risk of mortality from maternal complications. Lower Panel. Every pregnant woman is subject to a risk of developing major maternal complications, such as a sexually transmitted infection with chlamydia or gonorrhea, sepsis, postpartum hemorrhage, severe preeclampsia/eclampsia, or obstructed labor. Each maternal complication is associated with a further risk of death or long-term sequelae (e.g., infertility, severe anemia, neurological sequelae, rectovaginal fistula), which are associated with a decrement in health-related quality of life and costs related to either management or treatment.

The Maternal Health Policy Model is a computer-based state-transition model that simulates the natural history of pregnancy (both planned and unplanned) and pregnancy-related complications in a representative cohort of sexually-active Mexican women. Health states in the model reflect important characteristics that affect mortality, quality of life and resource use. (**Figure 1**) The time horizon incorporates a woman's entire lifetime and is divided into equal increments during which women transition from one health state to another. A cohort of 100 000 sexually-active 15-year-old girls enters the model, and faces a risk of becoming pregnant each year. The probability of becoming pregnant depends on a woman's age, history of pregnancy-related complications, and use of contraception. Women who become pregnant may experience a miscarriage or have an elective abortion, a fraction of which are unsafe (defined as a medically or surgically induced abortion performed by an untrained person), conferring a higher risk of death and complications. A woman who remains pregnant may have an uncomplicated course or she may develop a pregnancy- or delivery-related complication (e.g., severe preeclampsia/eclampsia, obstructed labor, postpartum hemorrhage, sepsis, sexually transmitted infection) associated with a risk of death and long-term sequelae (e.g., infertility, neurological sequelae, rectovaginal fistula, severe anemia). We assume that infertility can result following a sexually transmitted infection, unsafe abortion, or sepsis; women with severe anemia have a higher relative risk for death from maternal complications; and all women face age-specific risks of dying from other causes.

**Figure 2:**
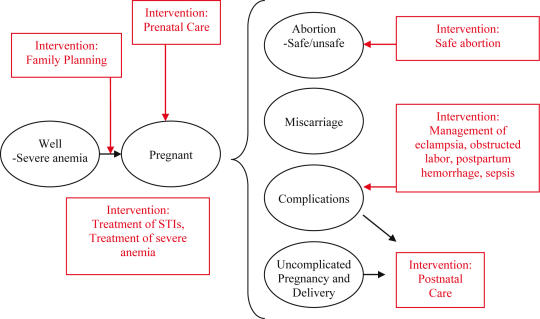
Schematic of Modeled Interventions. Interventions are applied to different points along the clinical course of pregnancy and delivery. Prenatal care, the treatment of sexually transmitted infections, and the management of severe anemia apply throughout the three trimesters of pregnancy prior to labor and delivery. Safe abortion applies to the first trimester of pregnancy. Hospital-based interventions such as the management of severe preeclampsia/eclampsia, obstructed labor, postpartum hemorrhage, and sepsis apply to the periods of labor and delivery as well as postpartum.

Interventions can be applied at different time points in a woman's reproductive life and pregnancy (**Figure 2**) and include: (1) provision of family planning; (2) safe abortion; (3) prenatal care (e.g., four to six prenatal visits including physical exam, urine protein screen, screening and treatment for anemia and syphilis, iron/folate supplementation, tetanus vaccination, and if indicated, treatment for sexually transmitted infections; (4) high-quality intrapartum care including access to skilled attendants *and* emergency obstetric care (e.g., timely access to a facility with surgical expertise, critical care capability including blood transfusions, and ability to manage serious obstetric complications that can cause death); and (5) postpartum care (e.g., postnatal visit including physical exam, iron and vitamin A supplementation).

The impact of an overall strategy is determined by the effectiveness of each individual intervention, the set of interventions included in the package of services, and the coverage achieved. (**Table 1**) When multiple interventions are evaluated within a single strategy, we assume interventions that target *different* maternal complications have an additive effect whereas those that act on the *same* maternal complication have a multiplicative effect. The effectiveness of family planning is estimated as a function of the performance and coverage level of the contraceptive method. The effect of other interventions is modeled as a reduction in the case fatality rate and/or morbidity risk of a specific maternal complication(s).

**Table 1:** Impact and coverage levels of interventions.*

Intervention	Impact on Mortality or Morbidity	Evidence Level†	Current Coverage in Mexico (%)	Recommended Coverage in MBP Standard of Care (%)
Family planning (<20 years)	Both	C	18	33
Family planning (≥20 years)	Both	C	59	74
Prenatal care	Uncertain	None	68	90
Treatment of severe anemia	Mortality	C	68	90
Treatment of symptomatic STI's	Morbidity	B	68	90
Skilled birth attendants	Mortality	D	86	90
Safe abortion	Both	B	50	‡
Management of complications				
Severe Preeclampsia/Eclampsia	Mortality	A	81	90
Obstructed labor	Both	A	81	90
Postpartum hemorrhage	Mortality	A	81	90
Sepsis	Mortality	A	81	90
Postpartum care	Uncertain	None	68	90

* MBP  =  Mother baby package; STI  = sexually transmitted infection

† Refers to whether direct or indirect evidence exists for the impact of the intervention on maternal mortality or morbidity. Evidence level is interpreted as follows for purposes of this analysis: *A* indicates that evidence comes from randomized controlled trial(s) conducted in a developed country setting, but the actual effectiveness of the intervention could be lower in developing countries due to reduced access and quality of care; *B* indicates that evidence comes from randomized controlled trial(s) conducted in a developed country setting, but actual intervention effectiveness is likely similar in developed and developing countries; *C* indicates that evidence is based on prospective cohort studies, observational and case control studies; *D* indicates an assumption is based solely on expert opinion; and *none* indicates an absence of evidence. The absence of evidence for an impact on mortality or morbidity is not intended to be interpreted as there is no effect, but indicates the absence of data to support an effect.

‡ Provision of safe abortion is not a component of the MBP. Since the MBP strategy represents an *upgrade* from current practice patterns, however, coverage of safe abortion remains at the current practice level of 50%.

In an initial analysis we simulate three scenarios to provide insight into the magnitude of what has been achieved by Mexico with the current standard of maternal care, and to assess the impact of upgrading selected strategies to the coverage levels recommended in the WHO *MBP*. In this analysis we use historical data to simulate natural history (i.e., absence of significant maternal care) in which coverage is assumed to be 14% for skilled birth attendance. We then simulate access to services and increased coverage levels over time to assess the incremental costs and benefits of the *current standard of care* in Mexico, in which coverage for primary level interventions is 68%, skilled attendance is 86%, the coverage of hospital-level interventions is 81%, and coverage for family planning is 59% in women 20 years of age and older and 18% for women under 20 years of age [10]–[13]. Finally, we comparatively assess an upgrade to the standard of care and coverage levels recommended in the *MBP* with a coverage of 90% for all interventions except family planning, whose coverage is increased from 59% to 74% in women age 20 and older, and from 18% to 33% in women younger than age 20 [4], [5].

In a second analysis, considered to be our base case analysis, we use the current *standard of care* in Mexico as the baseline comparator and assess the incremental cost-effectiveness of increasing coverage of individual interventions or subsets of interventions. We consider access to comprehensive *emergency obstetrical care (EmOC*) to be access to a facility with surgical expertise (e.g., cesarean section), critical care capability including blood transfusions, and ability to manage serious obstetric complications. However, we recognize ensuring high-quality intrapartum care will require different approaches and investments depending on the local situation and setting in the individual states within Mexico. Accordingly, we define *enhanced* access to comprehensive EmOC as an investment in any of the following: (a) a program to improve appropriate and timely referral rates; (b) improved transportation to facilitate rapid access to a hospital with a blood supply and surgical expertise; (c) new technologies to stabilize a woman en route to emergency care; (d) improved quality of health services for management of severe obstetrical complications.

### Model Input Data

A detailed summary of selected variable estimates and their plausible ranges is available in the **Appendix S1**. We estimated an annual rate of pregnancy under “natural fertility” conditions of 31% using data from Afghanistan, where abortion is illegal and access to modern contraception is limited [13]–[15]. In accordance with the 2004 Population Reference Bureau World Data, we assumed 68% of women ages 20–45 years use some method of birth control with 9% employing traditional methods (i.e., withdrawal or periodic abstinence) and 59% using modern methods (12% oral contraceptives, 6% condoms, 24% intrauterine devices, 5% injectable contraceptives, 51% sterilized, and 2% partners sterilized). We assumed a lower uptake of contraception among women <20 years age with 12.3% employing traditional methods and 17.7% using modern methods. We assumed 15% of all pregnancies result in a miscarriage (of which 33% require further management) [16], [17] and 17% end in abortion [18] (of which 50% are unsafe) [19], [20]. There is considerable uncertainty in estimates of unsafe abortion (in part due to underreporting), and thus we varied the risk of abortion, the proportion that are unsafe, and the abortion-related mortality rate in sensitivity analyses.

Because Mexico-specific data on the incidence of maternal complications and complication-related deaths are limited, we relied in part on regional estimates for Latin America and Caribbean region from the Global Burden of Disease (GBD) Study [21], [22]. The case fatality rate (CFR) for each maternal complication under “natural history” conditions was derived by assuming the “natural history” CFR was a function of the current CFR reported for Latin America and Caribbean, the maternal complication rate, and the coverage rate and effectiveness of applicable maternal health interventions [10]–[13], [21], [23]–[37]. Effectiveness data were from the published literature, using randomized controlled trials whenever possible, followed by prospective cohort studies and expert opinion [23]–[37]. When an intervention lacked a clear evidence base, we conservatively assumed there was no effect on mortality. Coverage rates for prenatal care, deliveries assisted by skilled attendants, and facility-based deliveries were taken from Mexico-specific estimates [10]–[13]. Risk for long-term complications, disability weights, and duration of disability were estimated from the GBD study [21].

Direct medical costs were based on a comprehensive study assessing the costs of interventions included in the WHO MBP in Morelos State, Mexico [4], [22]. Data included personnel, services, drugs, and both inpatient and outpatient services for three different levels of care: rural health center, urban health center, and hospital. Interventions such as treatment for severe anemia, treatment of sepsis, skill attendants, and family planning cost less when delivered in center-based settings compared with hospital-level settings. Costs related to the treatment of selected long-term complications associated with infertility, neurologic sequelae, and obstetric fistula were estimated using published studies conducted in other countries and scaled to approximate healthcare costs in Mexico [38]–[42]. Productivity costs associated with premature death were explored using sensitivity analysis [43], [44]. All costs were expressed in 2001 U.S. dollars. Also see **Appendix S1** for additional information.

### State-Specific Analyses

To provide insight into how maternal mortality trends differ by state, we grouped states into three categories using the “marginality index” constructed by the National Institute on Statistics and Geography, a composite index that considers nine indicators of socioeconomic status of the community (listed in the **Appendix S1**), and ranges from low (best-off) marginality to very high (worst-off) marginality [45]. Using the death registration records and the population statistics for year 2000 provided by the Ministry of Health in Mexico, we estimated disability-adjusted life years (DALY) attributable to the five Global Burden of Disease categories considered to be maternal conditions, by state and level of marginality. We then assessed the burden of maternal-related disease at the state level, conducted a time-trend analysis for a ten year period (1992–2002), and conducted subnational cost-effectiveness analyses.

## Results

### Model Performance and Face Validity

The model predicted a total fertility rate and crude birth rate of 2.7 births per woman and 21 births per 1,000 population compared to 2.6 births per woman and 21 to 24 births per 1,000 population reported for Mexico by UNICEF, WHO, and the Population Reference Bureau [11], [13], [46]. Model output (maternal deaths, live births) was used to generate a MMR for Mexico. After adjusting for the maternal complications included in the analysis, the model predicted an MMR of 85, closely approximating the MMR of 83 reported by the WHO for Mexico in 2000 [1].

### Clinical Outcomes, Costs, and Cost-Effectiveness Analysis

**Table 2** shows the percent reduction in mortality and morbidity associated with the current *standard of maternal care* (i.e., current effective coverage levels) and by upgrading selected strategies to coverage levels recommended in the *MBP standard of care,* compared with a historical scenario of no maternal care. Although the mortality reduction with the current standard of care in Mexico has been substantial, upgrading to the coverage levels in the *MBP standard of care* reduces the number of deaths for a cohort of 100,000 women, from 175 to 92, and cases of serious morbidity from 4,149 to 2,755, representing an approximate additional 50% reduction in mortality. In addition to being more effective than current practice, upgrading to the coverage levels in the *MBP standard of care* reduced the per-person lifetime costs from $503 to $372, and had an incremental cost-effectiveness ratio of $550 per life year saved (YLS) and $390 per disability-adjusted life year (DALY) averted.

**Table 2:** Benefits, costs, and cost-effectiveness of current practice in Mexico (compared with no maternal care), and upgrading to the coverage rates in the WHO Mother Baby Package (MBP) standard of care.*

Strategy	Mortality (# deaths per 100,000)	Morbidity (# events per 100,000)	Additional reduction in mortality vs. natural history, %	Additional reduction in morbidity vs. natural history, %	Costs (average discounted lifetime)	Life expectancy (average, discounted)	ICER ($/LY)	ICER ($/DALY)
Natural History	1,556	10,262	---	---	$237.16	28.4010	---	---
Current Practice in Mexico	175	4,149	88.7	59.6	$502.87	28.6321	†	†
MBP Standard of Care	92	2,755	94.1	73.2	$371.82	28.6463	550	390

* LY = Life years, DALY = Disability adjusted life years, ICER = incremental cost-effectiveness ratio; MBP = Mother Baby Package

† Current practice in Mexico (i.e., average coverage rates associated with status quo) is dominated by the coverage rates recommended in the MBP standard of care since the MBP is less costly and more effective. (see Methods for details)

**Table 3** shows the results of a second analysis in which we assessed the incremental benefits and cost-effectiveness of increasing coverage of individual interventions or subsets of interventions, compared to the current *standard of care* in Mexico. Each of the strategies shown was more effective and less costly than current practice. A combined approach of (1) increasing family planning from 59% to 74% in women age 20 and older, and from 18% to 33% in women younger than age 20, and (2) assuring access to safe abortion for *all* women who electively terminate a pregnancy, provided a 43% reduction in mortality and was cost saving relative to current practice. The most effective strategy added a third component to these two interventions, by (3) providing access to high-quality intrapartum care for all pregnant women and enhancing access to comprehensive emergency obstetric care for at least 90% of women. This strategy provided a 75% reduction in maternal mortality, a 47% reduction in morbidity, and had an incremental cost-effectiveness ratio of $300 per DALY averted relative to the next best strategy. Although other strategies shown were formally dominated by the provision of these three interventions, they were all cost saving relative to current practice in Mexico. For the 4 most effective strategies shown in **Table 3**, the cost savings over the lifetime of a cohort of 100,000 women would exceed $10 million.

**Table 3:** Maternal outcomes and cost-effectiveness of alternative strategies to improve maternal health compared with status quo in Mexico.*

Strategy	Mortality (# deaths per 100,000)	Morbidity (# events per 100,000)	Costs (average discounted lifetime)	Life expectancy (average discounted)	ICER ($/DALY)	Cost savings relative to current practice (per 100,000 women)†
*Current Practice in Mexico*	*175*	*4,149*	*$502.87*	*28.6321*	*---*	*---*
Current Practice plus **increased FP** (33%/74%), **safe abortion** (100%)	101 (43%)	2,261 (46%)	$386.23	28.6446	‡	$11,600,000
Current Practice plus **increased FP** (33%/74%), **safe abortion** (100%) and **enhanced IpC/EmOC** (100%/90%)	43 (75%)	2,204 (47%)	$390.21	28.6555	300§	$11,200,000
Current Practice plus **increased FP** (33%/74%), **enhanced IpC/EmOC** (100%/90%)	62 (64%)	2,769 (33%)	$391.30	28.6519	||	$11,100,000
Current Practice plus **increased FP** (33%/74%)	119 (32%)	2,825 (32%)	$397.30	28.6410	||	$10,500,000
Current Practice plus **safe abortion** (100%) and **enhanced IpC/EmOC** (100%/90%)	64 (64%)	3,241 (22%)	$493.78	28.6522	||	$900,000
Current Practice plus **enhanced IpC/EmOC** (100%/90%)	92 (48%)	4,068 (2%)	$495.03	28.6472	||	$800,000

* DALY = Disability adjusted life years, ICER = incremental cost-effectiveness ratio; IpC = intrapartum care; EmOC = emergency obstetric care; FP = family planning. Strategies increase coverage of specific interventions above the coverage rates in current practice. These include enhanced high-quality intrapartum care for all pregnant women (81% to 100%) and enhancing access to comprehensive emergency obstetric care for at least 90% (81% to 90%), safe abortion (from 50% to 100%), and FP (from 59% to 74% in women age 20 and older, and from 18% to 33% in women younger than age 20). All strategies are compared to current coverage; incremental cost-effectiveness ratios are assessed by ranking the strategies from the least costly to most costly and calculating the incremental change in costs and benefits compared to the next best strategy. For strategies that include enhanced IpC/EmOC access we assumed an incremental cost of $18.50 per woman requiring referral. Also see results section.

† Cost savings relative to current practice (per 100,000 women) is an indicator of the resources that would be saved over the lifetime of a cohort of 100,000 women relative to current practice in Mexico if a particular strategy was adopted. This savings is calculated as the difference in total lifetime costs for a strategy compared to current practice, multiplied by 100,000.

‡ Increased family planning (74% in women age 20 and older, 33% in women younger than age 20) with increased safe abortion (100%) is more effective and less costly than current practice in Mexico.

§ Increased family planning (74% in women age 20 and older, 33% in women younger than age 20) with increased safe abortion (100%) and enhanced IpC/EmOC access (100%/90%) has a cost-effectiveness ratio of $300/DALY compared to the next best strategy of increased family planning with increased safe abortion alone.

|| Strategy is less effective and more costly than increased family planning (74% in women age 20 and older, 33% in women younger than age 20) with increased safe abortion (100%) and enhanced IpC/EmOC access (100%/90%) and is therefore formally dominated. Compared to current practice, these strategies are still cost saving.

### Sensitivity Analysis

Cost-effectiveness results were most sensitive to the cost of increasing coverage of interventions above and beyond the current practice in Mexico. The magnitude of the reduction in maternal mortality was most sensitive to the assumptions about the baseline effective coverage rates assumed in the base case, the risk of mortality due to unsafe abortion, and the effectiveness of maternal health interventions.

**Figure 3:**
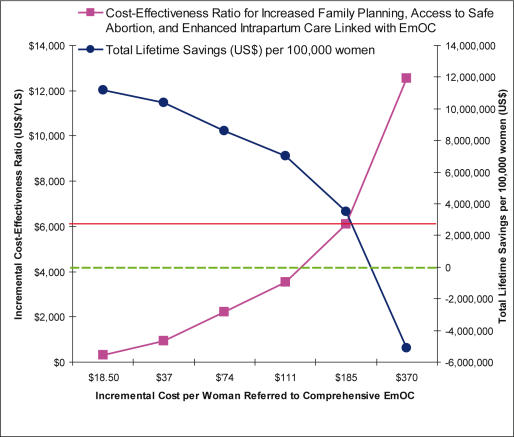
The Impact of Costs Invested in Enhancing Access to EmOC. The additional costs required to *enhance* access to comprehensive EmOC, expressed as the composite cost of a *successfully referred* woman, is assumed to include the costs required for ensuring recognition of the need for referral, expedient transport, and ultimate access to an appropriate facility capable of comprehensive EmOC. Shown is the impact of varying the cost per successfully referred woman from $18.50 to $370, on the incremental cost-effectiveness ratios (ICER) for a strategy that includes (1) an increase in family planning from 59% to 74% in women age 20 and older, and from 18% to 33% in women younger than age 20, (2) access to safe abortion for *all* women who electively terminate a pregnancy; and (3) access to high-quality intrapartum care for all pregnant women and *enhanced access* to comprehensive emergency obstetric care for at least 90% of women (pink line), compared with a strategy only focusing on family planning and safe abortion. Also shown is the impact on the total lifetime savings for a cohort of 100,000 women that could be achieved using this strategy as compared to *current practice* in Mexico (blue line). Provided the incremental cost was below $120 per *successfully referred* woman, the most effective strategy would be associated with a lower average per-woman lifetime cost than that of current practice (green dashed line). Even at a cost of $185 per successfully referred woman (red solid line), the incremental cost-effectiveness ratio was less than the Mexico-specific GDP per capita, and would be considered very cost-effective.

**Figure 3** shows how the incremental cost-effectiveness ratio associated with the most effective strategy changes as we vary the additional costs associated with providing *enhanced* access to comprehensive emergency obstetric care. We express this as a composite cost of a *successfully referred* woman, and assume it includes the costs required for ensuring recognition of the need for referral, expedient transport, and ultimate access to an appropriate facility capable of comprehensive EmOC. We estimate that up to 18.5% of pregnant women ultimately need emergency care, from 20% to 30% will be referred, and vary the cost per successful referral from $18.50 (base case) to $370. Provided the incremental cost was below $120 per *successfully referred* woman, the most effective strategy would be associated with a lower average per-woman lifetime cost than that of current practice. Even at a cost of $185 per successfully referred woman, the incremental cost-effectiveness ratio was below the Mexico-specific Gross Domestic Product (GDP) per capita ($6,172) [47], and would therefore be considered very cost-effective [48].

Because of the uncertainty in the underlying parameters and assumptions around abortion, we conducted a sensitivity analysis in which we varied the case fatality rate of unsafe abortion, the underlying rate of abortion among pregnant women, and the proportion of abortion that is unsafe. Under base case assumptions, the incremental cost-effectiveness ratio associated with provision of safe abortion for all women desiring elective termination of pregnancy, but with no other changes or improvements in any dimension of safe motherhood relative to standard care, was approximately $1,400 per YLS, less than 25% of the GDP per capita. If the underlying rate of abortion in a pregnant woman is increased by 1.5, the case fatality rate due to unsafe abortion is increased to 0.002 or greater, the proportion of unsafe abortion to safe abortion is increased by 12.5%, and/or if the rate of attributable morbidity and costs of that morbidity are more than 2 times higher, the incremental cost-effectiveness ratio associated with provision of safe abortion is less than 10% of the GDP and in many cases less than 5% of the GDP (corresponding to cost-effectiveness ratios of $100 to $500 per YLS or DALY averted).

### State-Specific Analyses

When analyzed over ten years (1992 to 2002), the reduction in maternal mortality rate was 24.4% for high marginality states, 18.9% for medium marginality states, and essentially unchanged for low marginality states. Results of our cost-effectiveness analyses repeated for low and high marginality states showed very similar results to the base case, although the cost savings over the lifetime of a cohort of 100,000 women ranged from $12.3 to $13 million in high marginality states and $8.7 to $8.9 million in low marginality states.

Supplementary results for selected strategies are provided in the **Appendix S1.**

## Discussion

We developed a flexible decision analytic policy model to simulate a population of women through their childbearing years, calibrated the model to country-specific data in Mexico, and conducted a comparative policy analysis to identify the potential clinical benefits and cost-effectiveness of alternative strategies for maternal death and disability reduction in Mexico. Our analysis extends the work of others [37], [49]–[53] by leveraging primary national and state-level data available in Mexico, adopting an analytic approach that considers the risks associated with pregnancy over a woman's entire lifetime, and including safe abortion among the important interventions to consider as part of a comprehensive strategy to reduce maternal morbidity and mortality.

The results of our analysis suggest several strategies would improve maternal health in Mexico and be cost-effective. Although the mortality reduction with the current coverage levels for maternal health interventions in Mexico has been substantial when compared to the national situation several decades ago, incremental improvements in coverage levels to those recommended in the *MBP standard of care*, specifically for family planning and provision of high-quality intrapartum/obstetric care, provide greater health benefits *and* save resources. Although the overall reduction in maternal mortality between 1992 and 2002 in Mexico approximated only 22%, it was far greater in high- and medium-marginality states than low-marginality states, indicating that national efforts were successful in reducing disparities in safe motherhood within the country.

We also identified several additional strategies involving one or more incremental improvements to current practice in Mexico that were capable of providing substantial clinical benefits as well as cost savings. Among these options, the most effective strategy consisted of three enhancements to current practice: (1) increasing family planning from 59% to 74% in women age 20 and older, and from 18% to 33% in women younger than age 20; (2) ensuring access to safe abortion for women electing to terminate a pregnancy; and (3) providing access to intrapartum care for all pregnant women and enhancing access to comprehensive emergency obstetric care for at least 90% of women. This was the only strategy with enough of an impact to attain the target of 75% maternal mortality reduction set by Millennium Development Goal 5.

Also worthy of serious consideration are single- or paired-intervention strategies involving enhancements in the areas of family planning, safe abortion, or emergency obstetric care. Although none of these strategies was ever as attractive as the package of improvements (family planning, safe abortion and access to emergency obstetric care), they each would provide comparable or greater benefit than that made by Mexico on average in the last decade. Moreover, nearly all strategies would result in cost-savings over the long-term relative to current practice. The only exception is the single intervention strategy of enhanced safe abortion, which, while not cost-saving, would still be considered highly cost-effective since it has an incremental cost-effectiveness ratio that is well below Mexico's GDP per capita of $6,172 [47]. Of note, a combined approach that improves access to safe abortion and increases effective coverage for family planning is synergistic in reducing unwanted pregnancies, reducing maternal morbidity and mortality, and increasing health returns for investments using public health dollars. The cost savings from providing these two interventions together, over the long-term, exceeds $11 million per 100,000 women of reproductive age followed over their lifetime.

While interventions that improve health at a cost should ideally be compared with other interventions that compete for the same resources, there is no universal criterion that defines a threshold cost-effectiveness ratio, below which an intervention would be considered cost-effective. A commonly cited rule of thumb is based on a report by the Commission on Macroeconomics and Health, following which others suggested that interventions are “very cost-effective” and “cost-effective” if they have cost-effectiveness ratios less than per capita GDP or three-times the per capita GDP, respectively [48]. Given that nearly all strategies we found to be most effective were also associated with incremental cost-effectiveness ratios that were only a fraction of Mexico's GDP per capita of $6,172 [47], investments in improving maternal health are likely to be one of the most cost-effective interventions that could be implemented in Mexico.

Over a short time horizon, additional costs will be required to implement many of the strategies we identified as cost-effective. In fact, in the published costing study conducted in Morelos state, the total cost of upgrading to the WHO Mother Baby Package standard of care was estimated to be twice that of the current standard of care [4]. While a costing study provides useful information for defining current budgetary needs, a cost-effectiveness analysis aims to identify health investments that provide the best value given some resource constraint over a long time horizon. Adopting the latter perspective, we identified several approaches that would save lives, reduce morbidity, and save monetary resources compared with current practice. For example, using current census data from Mexico, our most effective strategy that included enhanced family planning and safe abortion as two of its three main components, would save approximately $116 million on average, over the lifetime of a single birth cohort. The savings in high-marginality states ($49,572,000) would be greater than those with medium marginality ($39,579,000) or lower marginality ($26,530,000). While additional short-term funds might be needed, this analysis provides valuable information to the Ministry of Health assessing where investment of the next dollar would make the most difference.

Recently, there has been a published policy statement supporting implementation of “effective intrapartum care” as first priority, followed by family planning and safe abortion as complementary strategies, as the strategic approach with the best chances of reducing maternal mortality in developing countries [54]. In addition, attention has been focused on the inclusion of safe abortion care interventions (which include access to safe abortion, treatment of abortion complications, and provision of post-abortion contraception) as a primary strategy in addition to EmOC for maternal mortality reduction [55], [56]. While our results support the general principles behind these recommendations, maternal mortality in Mexico is relatively low in comparison to other developing countries, and this is likely attributable to a relatively high coverage of, and access to, intrapartum care. Our analysis indicates that while further improvements in the intrapartum period, and particularly enhanced access to high-quality comprehensive emergency obstetrical care, will clearly prevent maternal deaths, a substantial impact at the state and national level could be realized by a special focus and effort on family planning in women of all ages and safe abortion.

Our study has several limitations. There are many data gaps and the quality of available information was variable. We limited our analysis to long-term sequelae for which data were accessible and therefore may have underestimated the burden of disease related to pregnancy and childbirth in Mexico. We focused on the interventions included in the WHO Mother Baby Package (*MBP*) plus access to safe abortion, in large part because data were available for many of these. There are others, such as improvements in the management of hypertensive disorders of pregnancy and enhanced care for women at high-risk because of co-morbidities, such as diabetes, that were not included. An advantage of a modeling approach in which a durable tool is developed and calibrated to a country-specific situation, is that as better data become available, results may be expediently refined. These two specific examples will be important data gaps to remedy.

Second, programmatic costs above and beyond the resources reflected in the direct medical cost estimates were not included as they will likely be state-specific and data are not currently available. Accordingly, priority areas to focus data collection efforts include the costs of (1) alternative delivery strategies for family planning and safe abortion, (2) training the provider base for provision of safe abortion, (3) strategies to improve the quality of obstetric care, and (4) scaling-up access to comprehensive EmOC. In general, high marginality states will have greater resource requirements in terms of overcoming the human resource and training barriers, as well as in creating the access and infrastructure necessary to provide improvements in maternal health strategies, and should be the first priority. In addition, the resources required for education, advocacy, overcoming cultural barriers, and political mobilization, all of which would be relevant for increasing access to safe abortion for example, are not all monetary, and are certainly complex. That being said, analyses such as this one, which demonstrate the substantial cost savings and health gains with safe motherhood interventions, may help to mobilize political support and advocate for societal change.

Our results may not be generalizable to other countries due to heterogeneity in risk for poor maternal outcomes, differences in existing health infrastructure, and differences in the presence and severity of monetary and nonmonetary constraints [57], [58]. Although the qualitative themes we identified are likely to be robust across regions, operational approaches to delivering care and strategies to reach the poorest women will likely vary in different settings. Although future work will expand the choice set of interventions to include neonatal outcomes, we purposefully chose to focus on strategies to reduce maternal mortality and morbidity in this analysis. Although neonatal outcomes were not quantified, their inclusion would only make these results more attractive.

There are several strategic options to improve maternal health in Mexico that would narrow disparities between states and be cost-effective. Increasing the provision of family planning above current coverage levels and assurance of access to safe abortion for all women are complementary and cost-effective strategies that will provide the greatest benefit within a short-time frame. In the long-term, aggressive efforts to implement a dually-focused strategy that reduces both the unmet need for family planning *and* the risk of unsafe abortion, saves lives and substantial monetary resources. Incremental improvement in access to high-quality intra-partum care within, or with functional rapid linkages to, a setting able to deliver high-quality care to manage obstetric emergencies will further reduce maternal deaths and disability. While these strategies will require additional short-term financial resources, they will save societal resources in the long-term.

## Supporting Information

Appendix S1 (0.57 MB DOC)
