# Supplementary material for: The Costs, Benefits, and Cost-Effectiveness of Interventions to Reduce Maternal Morbidity and Mortality in Mexico
Source: PLoS One. 2007 Aug 15;2(8):e750. doi: 10.1371/journal.pone.0000750 (PMC1939734; doi:10.1371/journal.pone.0000750)
Supplement: Appendix S1 — (0.57 MB DOC) [file pone.0000750.s001.doc]

**SUPPLEMENTAL APPENDIX**

**A.1 SUPPLEMENTAL NATURAL HISTORY INFORMATION:**

A.1 Assumptions about Maternal Complication-related Case-Fatality and Disability Rates

Based on our consultation with experts, the case fatality rate (CFR) for each maternal complication under “natural history” conditions (i.e., absence of significant maternal care) was derived by assuming the CFR was a function of: the risk of developing a given maternal complication; the proportion of attributable maternal deaths to new cases of a given maternal complication (for Latin America and Caribbean region from the Global Burden of Disease (GBD) study); and the coverage rate and effectiveness of applicable maternal health intervention(s) [1-22]. In this way, we estimated the case-fatality rate for severe pre-eclampsia/eclampsia (0.75%), obstructed labor (1.33%), sepsis (1.32%), and postpartum hemorrhage (0.92%).

The risk of acquiring a disability for a given maternal complication was calculated by dividing the incidence of a given disability by the incidence of the attributable maternal complication as reported in the GBD study for Latin America and the Caribbean region [2]. This method was used to estimate the risk of neurological sequelae (1.00%) from severe preeclampsia/ eclampsia, the risk of rectovaginal fistula (3.45%) from obstructed labor, the risk of Sheehan’s syndrome (0.80%) and severe anemia (0.96%) from postpartum hemorrhage, and infertility (22.1%) from sepsis.

**A.2. SUPPLEMENTAL COST INFORMATION:**

A.2.1 Assumptions about the Cost of Maternal Health Interventions

Direct medical costs for interventions within the World Health Organization’s (WHO) Mother Baby Package (MBP) were estimated from a published costing study of the Mother Baby Package applied to Morelos state, Mexico [22], a related unpublished report [3], and the WHO’s MBP costing spreadsheet [23]. For each intervention, costs under the current standard of care and for upgrading to or beyond the coverage levels and standard of care for the MBP were reported for three different levels of care: rural health center, urban health center, and hospital. Using questionnaires, detailed information on maternal health interventions was retrieved from medical personnel in 2 rural health centers, 1 urban health center, and 1 general hospital. Retrieved information for each intervention included: type and dose of drugs; required supplies; qualifications of personnel in the facility; time dedicated to each patient by personnel; and length of hospital stay. Intervention costing inputs included the cost of blood products, drugs, supplies, hospital bed per day, personnel time, and emergency transportation (gasoline). The costs of drugs and medical supplies were drawn from a price list provided by the Morelos State Department of Health and supplemented by prices reported by UNICEF when local estimates were not available. Using primary data provided by the study’s authors on the utilization patterns of individual interventions for each level of care, average costs for interventions were estimated for the current and MBP standard of care.

Several interventions were estimated to cost less under the MBP standard of care compared to current practice and include: (1) management of severe anemia, (2) normal delivery with skilled attendant, (3) postnatal care, (4) treatment of sepsis, and (5) elements of family planning such as oral contraceptives, condoms, and sterilization. The cost reduction related to these interventions is due to assumptions about the shift from hospital-level care to center-based care for these interventions and the associated decrease in hospital-level personnel costs, drugs, and consumable supplies.

All costs were expressed in 2001 U.S. dollars. For interventions within the Mother Baby Package, the Morelos State study reported costs in 2001 U.S. dollars and used an exchange rate of 9.30 pesos per dollar to transform costs from Mexican to U.S. currency. For unsafe abortion, costs were inflated to reflect 2001 U.S. dollars using the Consumer Price Index.

A.2.2 Assumptions about the Cost of Maternal Morbidity

Direct medical costs related to the treatment of major maternal morbidity (i.e., infertility, neurological sequelae, Sheehan’s syndrome, and fistula) resulting from obstetric complications were estimated from a variety of data sources from different countries due to the absence of Mexico- or Latin America-specific estimates. The ratio of the gross domestic product (GDP) per capita of Mexico to the study country was used as a multiplier by which to scale estimates to approximate their cost in Mexico. When necessary, costs were further inflated or deflated to reflect 2001 U.S. dollars. For example, the cost of fistula repair was based on a report from EngenderHealth, which estimated the cost of fistula repair to be $300-$400 in Africa [24]. For use in the Mexico-specific analysis, this cost was scaled to reflect the differential in GDP per capita between Mexico and Nigeria. When necessary, costs were further inflated or deflated to reflect 2001 U.S. dollars using the Consumer Price Index.

Because of a lack of cost information specific to neurological sequelae resulting from severe preeclampsia/eclampsia and Sheehan’s syndrome, we relied on cost studies for the treatment of stroke and hypopituitarism [25,26]. We conservatively assumed 25% of women with infertility or fistula sought curative medical treatment. In the absence of data to inform the yearly cost of unrepaired fistula, we assumed a cost of $500 per year based on consultation with experts. Sensitivity analyses were conducted varying the proportion of women pursuing curative treatment and the costs of treated and untreated complications.

Appendix Table 1. Selected Model Parameters.*

| Variable | | | | | Base Case | Plausible Range | Sources |
| --- | --- | --- | --- | --- | --- | --- | --- |
| **Natural History Parameters** | | | | | | |  |
|  | Pregnancy | | | | 0.310 | 0.100-0.400 | 1,27,28 |
|  | Miscarriage | | | | 0.150 | 0.120-0.310 | 29,30 |
|  |  | Incomplete abortion | | | 0.330 | † | 29,30 |
|  | Sexually transmitted infection (gonorrhea or chlamydia) | | | | 0.100 | † | 2,3 |
|  |  | Symptomatic lower genital tract infection | | | 0.250 | † | 31 |
|  |  |  | Acute PID, among untreated/uncured | | 0.300 | 0.004-0.400 | 31-33 |
|  |  |  |  | Symptomatic acute PID | 0.400 | 0.150-0.400 | 31,34 |
|  |  |  |  | Infertility | 0.250 | 0.110-0.450 | 2 |
|  | Severe preeclampsia/eclampsia | | | | 0.005 | † | 2,3 |
|  | Obstructed labor | | | | 0.050 | † | 2,3 |
|  | Postpartum hemorrhage | | | | 0.050 | † | 2,3 |
|  | Sepsis | | | | 0.080 | † | 2,3 |
|  | Severe anemia | | | | 0.020 | † | 2,3 |
|  |  | Relative risk death from complication | | | 3.51 | ‡ | 35 |

| **Appendix Table 1. Selected Model Parameters. (cont.)***** **Intervention Parameters**   | | Variable | Base Case | Plausible Range | Sources | | --- | --- | --- | --- | | | --- | --- | --- | --- | --- | | | | | | | | |
| --- | --- | --- | --- | --- | --- | --- | --- | --- | --- | --- | --- | --- |
|  | Any contraceptive method | | | | 0.680 | ‡ | 1 |
|  |  | Traditional methods | | | 0.090 | ‡ | 1 |
|  |  |  | Failure rate | | 0.184 | † | 36,37 |
|  |  | Modern methods | | | 0.590 | ‡ | 1 |
|  |  |  | Oral contraceptives | | 0.120 | † | 1 |
|  |  |  |  | Failure rate | 0.069 | ‡ | 36,37 |
|  |  |  | Condom | | 0.060 | ‡ | 1 |
|  |  |  |  | Failure rate | 0.098 | † | 36,37 |
|  |  |  | Intrauterine device | | 0.240 | ‡ | 1 |
|  |  |  |  | Failure rate | 0.018 | † | 36,37 |
|  |  |  | Injectable contraceptives | | 0.050 | ‡ | 1 |
|  |  |  |  | Failure rate | 0.029 | † | 36,37 |
|  |  |  | Female sterilization | | 0.510 | ‡ | 1 |
|  |  |  |  | Failure rate | 0.005 | † | 36,37 |
|  |  |  | Male sterilization | | 0.020 | ‡ | 1 |
|  |  |  |  | Failure rate | 0.002 | † | 36,37 |
|  | Induced abortion | | | | 0.170 | † | 38 |
|  |  | Safe abortion | | | 0.085 | † | 39,40 |
|  |  |  | Death | | 0.000006 | † | 41,42 |
|  |  | Unsafe abortion | | | 0.085 | † | 39,40 |
|  |  |  | Infertility | | 0.030 | † | 2 |
|  |  |  | Death | | 0.001 | † | 40,41 |

| ***Appendix Table 1. Selected Model Parameters. (cont.)****  |  | | | | | --- | --- | --- | --- | | **SourcesVariable** | | | | | Base Case Plausible Range |  |  |  | | | | | | | | |
| --- | --- | --- | --- | --- | --- | --- | --- | --- | --- | --- | --- | --- | --- | --- | --- | --- | --- | --- | --- |
| **Intervention Parameters** | | | | | | | |
|  | Sexually transmitted infection (STI) with gonorrhea or chlamydia | | | |  |  |  |
|  |  | | Effectiveness of antibiotics for lower genital tract infection§ | | 0.960 | 0.940-1.00 | 4 |
|  |  | Effectiveness of treatment for acute PID|| | | | 0.600 | ‡ | 5-7 |
|  | Treatment of severe preeclampsia/eclampsia | | | |  |  | |
|  |  | Risk reduction for maternal death | | | 0.590 | ‡ | 8-10,18 |
|  | Management of obstructed labor | | | |  |  | |
|  |  | Risk reduction for maternal death | | | 0.950 | ‡ | 11-14,18 |
|  | Treatment of postpartum hemorrhage | | | |  |  | |
|  |  | Risk reduction for maternal death | | | 0.750 | ‡ | 15,18 |
|  | Sepsis** | | | |  |  |  |
|  |  | | | Risk reduction for maternal death with treatment | 0.900 | ‡ | 16-18 |
|  |  | | | Risk reduction for maternal death with  skilled attendance | 0.400 | ‡ | 18 |

| **Cost Parameters (2001 $US)** | | | | | | |  |
| --- | --- | --- | --- | --- | --- | --- | --- |
|  | Variable | | Base Case | MBP Standard | Sources | | |
|  | Family Planning (cost per year) | |  |  | | |  |
|  |  | Oral contraceptives†† | 14.23 | 13.08 | | 3 |  |
|  |  | Condoms†† | 27.10 | 14.79 | | 3 |  |
|  |  | Intrauterine device | 7.88 | 14.56 | | 3 |  |
|  |  | Injectable contraceptives | 17.26 | 18.63 | | 3 |  |
|  |  | Sterilization†† | 30.98 | 30.78 | | 3 |  |
|  | Abortion | |  |  | | |  |
|  |  | Safe abortion and incomplete abortion | 65.73 | 136.90 | | 3 |  |
|  |  | Unsafe abortion | 150.00 | 150.00 | | 43 |  |
|  | Prenatal care | | 40.48 | 49.76 | | 3 |  |
|  | Management of severe anemia†† | | 14.53 | 9.94 | | 3 |  |

| **Appendix Table 1. Selected Model Parameters. (cont.)***** | | | | | |
| --- | --- | --- | --- | --- | --- |
| Variable | | | Base Case | MBP Standard | Sources |
| **Cost Parameters (2001 $US)** | | | | | |
|  | Treatment of sexually transmitted infection†† | | 6.51 | 8.73 | 3 |
|  | Normal delivery with skilled attendant†† | | 129.61 | 73.28 | 3 |
|  | Management of severe preeclampsia/ eclampsia‡‡ | | 575.65 | 583.82 | 3 |
|  | Management of obstructed labor‡‡ | | 260.51 | 265.70 | 3 |
|  | Management of postpartum hemorrhage‡‡ | | 218.09 | 223.55 | 3 |
|  | Postnatal care†† | | 8.27 | 3.44 | 3 |
|  | Management of sepsis††,‡‡ | | 102.96 | 82.20 | 3 |
| Variable | | | Base Case | Plausible Range | Sources |
| **Costs of Long-Term Sequelae** | | |  |  |  |
|  | Management of infertility | | 65.80 | 32.90-164.50 | 44 |
|  | Treatment of PID | | 128.40 | 65.20-321.00 | 45 |
|  | Rectovaginal fistula | |  |  |  |
|  |  | Fistula repair | 3,000 | 300-18,000 | 24 |
|  |  | Unrepaired fistula (per year) | 500 | 50-3000 |  |
|  | Management of Sheehan’s syndrome | | 353.20 | 176.60-883.00 | 25 |
|  | Management of neurological sequelae | |  |  | |
|  |  | First year | 6254.40 | 3127.20-15,636 | 26 |
|  |  | Subsequent years (per year) | 2779.80 | 1389.90-6949.50 | 26 |

| **Appendix Table 1. Selected Model Parameters. (cont.)***** | | | | |
| --- | --- | --- | --- | --- |
| Variable | | Base Case | Duration of Effect | Sources |
| **Quality of Life Weights** | | | | |
|  | Infertility | 0.180 | Until age 45 | 2 |
|  | Severe anemia | 0.065 | 1 year | 2 |
|  | Neurological sequelae§§ | 0.388 | Lifetime | 2 |
|  | Rectovaginal fistula | 0.430 | Lifetime | 2 |
|  | Sheehan’s syndrome§§ | 0.093 | Lifetime | 2 |
| * | PID = pelvic inflammatory disease. | | | |
| † | Base case values were varied from 50% – 200% in sensitivity analysis | | | |
| ‡ | Base vase values were varied from 25% – 150% in sensitivity analysis | | | |
| § | For both gonorrhea and chlamydia, treatment includes a 7-day oral regimen of erythromycin (250mg four times a day) and a single, intramuscular injection of ceftriaxone (250mg). | | | |
| || | Treatment regimens vary and assume 80% receive outpatient treatment and 20% require inpatient treatment. | | | |
| ** | Treatment regimen includes a 2-day intravenous course of ampicillin, gentamycin, and metronidazole followed by an 8-day course of intramuscular gentamycin and oral metronidazole. | | | |
| †† | These interventions were estimated to cost less under the *MBP standard of care* compared to *current practice* due to assumptions about the shift from hospital-level care to center-based care and the associated decrease in hospital-level personnel costs, drugs, and consumable supplies. | | | |
| ‡‡ | Management of severe complications, near and during active labor, depends on access to a facility capable of comprehensive emergency obstetric care. Cost shown does not reflect the incremental costs of either transportation to the facility or temporizing care/technology to reduce death risk en route to an appropriate facility. For strategies that include enhanced access to EmOC we express this as a composite cost of a *successfully referred* woman, and assume it includes the costs required for ensuring recognition of the need for referral, expedient transport, and ultimate access to an appropriate facility capable of comprehensive EmOC. | | | |
| §§ | Variable is age-dependent, and value shown is for women ages 15-44 years old. | | | |

**B. SUPPLEMENTAL INFORMATION ABOUT THE MARGINALITY ANALYSES:**

A full report of this analysis is being prepared as a separate manuscript but the following text is provided to facilitate the reader’s understanding of the estimates used in the base case.

B.1 Marginality Index and Assignment of Marginality Grades to States

The “marginality index” as constructed by the National Institute on Statistics and Geography, is a composite index estimated at the level of the municipality and the state that considers nine indicators of socioeconomic status of the community [46]**.**

**Appendix Table 2.** **Components of the Marginality Index in Mexico**

| % population aged >15 years who are illiterate |
| --- |
| % population aged >15 years who have not completed primary school |
| % population living in dwellings without sewage and private toilet |
| % population living in dwellings without electricity |
| % population living in dwellings without piped water |
| % population living in dwellings that are crowded |
| % population living in dwellings with dirt floor |
| % population living in counties with <5,000 inhabitants |
| % population earning up to 2 times the min wage |

This index is used within Mexico for sub-national analysis of health, and assigns states into one of five categories, ranging from very low (best-off) marginality to very high (worst-off) marginality.

We classified maternal deaths by marginality of the state of residence and then regrouped the country into three categories of marginality of approximately equal numbers of women (approximately 10 to 11 million) of reproductive age (15-49 years old).

**Appendix Table 3.** **States re-categorized into 3 zones of marginality**

| **State Name** | **Marginality Grade** |
| --- | --- |
| Chiapas | High |
| Oaxaca | High |
| Guerrero | High |
| Veracruz | High |
| Hidalgo | High |
| **Appendix Table 3.** **States re-categorized into 3 zones of marginality (cont.)** | |
| **State Name** | **Marginality Grade** |
| Puebla | High |
| Campeche | High |
| Tabasco | High |
| San Luis Potosi | High |
| Yucatan | High |
| Michoacan | High |
| Zacatecas | Medium |
| Guanajuato | Medium |
| Nayarit | Medium |
| Tlaxacla | Medium |
| Sinaloa | Medium |
| Durango | Medium |
| Queretaro | Medium |
| Morelos | Medium |
| Quintana Roo | Medium |
| Estado de Mexico | Medium |
| Colima | Medium |
| Tamaulipas | Medium |
| Jalisco | Low |
| Sonora | Low |
| Chihuahua | Low |
| Baja California Sur | Low |
| Aguascalientes | Low |
| Coahuila | Low |
| Baja California | Low |
| Nuevo Leon | Low |
| Distrito Federal | Low |

B.2 Differences in Attributable DALYs and Maternal Mortality Rate, by Marginality

Using the death registration records and the population statistics for year 2000 provided by the Ministry of Health in Mexico, we estimated DALYs attributable to the five Global Burden of Disease categories considered to be maternal conditions. We then estimated DALYs attributable to the five Global Burden of Disease categories considered to be maternal conditions by level of marginality. In Appendix Figure 1, we show simple examples from very high (Upper Panel) and very low (Lower Panel) marginality states.

**Appendix Fig. 1.** (Upper Panel) Total DALYs by marginality per 10,000 women for the year 2002.

**Appendix Fig. 1.** (Lower Panel) Total DALYs by marginality per 10,000 women for the year 2002.

Since the death certificates contained information on state of residence (sub-divided into rural, semi-urban, or urban), we were able to classify maternal deaths by marginality of the state of residence, and analyze trends by socioeconomic status of the community historically for the time period from 1992 – 2002. We conducted a trend analysis to discern whether trends over time, by region, would cause a significant rank-order change, and it did not. From the same death records that were used to calculate the initial DALY burden, and using 3-year floating averages to smooth temporal trends, we plotted number of deaths per 10,000 women, for each of the new 3 zone marginality regions. Results from our initial analyses suggest that differences in maternal outcomes do differ in states with different marginality scores. We found that consistently, those regions categorized as having high marginality (being worse off) had a higher death rate per 10,000 women aged 15-49, ranging from .532 - .738 compared to .468-.577 for medium marginality, and .341-.388 for low marginality. When analyzed over time, states with the highest maternal burdens also exhibited a reduction in mortality each year, while those states in lower burden areas remained fairly flat.

## **Appendix Table 4: State-specific results for selected strategies (high and low marginality states)***

| Strategy | | Reduction in mortality vs. current practice, % | Reduction in morbidity vs. current practice, % | Costs, $  (average discounted lifetime) | ICER ($/YLS) | ICER ($/DALY) | Cost savings relative to current practice (per 100,000 women)† |
| --- | --- | --- | --- | --- | --- | --- | --- |
| **High Marginality States** | | | | | | | |
| Current practice in Mexico | | --- | --- | 508.21 | --- | --- | --- |
| Current Practice plus **increased FP** (33%/74%), **safe abortion** (100%) | | 44.8 | 48.4 | 378.11 | ‡ | ‡ | 13,009,000 |
| Current Practice plus **increased FP** (33%/74%), **safe abortion** (100%), and **enhanced IpC/EmOC** (100%/90%) | | 78.0 | 49.9 | 384.03 | 470**§** | 390**§** | 12,418,000 |
| **Low Marginality States** | | | | | | | |
| Current practice in Mexico | | --- | --- | 490.28 | --- | --- |  |
| Current Practice plus **increased FP** (33%/74%), **safe abortion** (100%) | | 37.9 | 39.3 | 401.32 | ‡ | ‡ | 8,896,000 |
| Current Practice plus **increased FP** (33%/74%), **safe abortion** (100%), and **enhanced IpC/EmOC** (100%/90%) | | 68.1 | 40.4 | 401.68 | 50**§** | 40**§** | 8,860,000 |
| * | DALY = Disability adjusted life years, ICER = incremental cost-effectiveness ratio; IpC = intrapartum care; EmOC = emergency obstetric care; FP = family planning. Strategies increase coverage of specific interventions above the coverage rates in current practice. These include enhanced high-quality intrapartum care for all pregnant women (81% to 100%) and enhancing access to comprehensive emergency obstetric care for at least 90% of women (81% to 90%), safe abortion (from 50% to 100%), and FP (from 59% to 74% in women age 20 and older, and from 18% to 33% in women younger than age 20). All strategies are compared to current coverage; incremental cost-effectiveness ratios are assessed by ranking the strategies from the least costly to most costly and calculating the incremental change in costs and benefits with the next best strategy. For strategies that include enhanced IpC/EmOC access we assumed an additional cost of $18.50 per woman requiring referral. Medium marginality states assume rates of maternal mortality and morbidity, and coverage of interventions, that approximate data reported for national average rates, and are shown as the base case results in the main manuscript. | | | | | | |
| † | Cost savings relative to current practice (per 100,000 women) is an indicator of the resources that would be saved over the lifetime of a cohort of 100,000 women relative to current practice in Mexico if a particular strategy was adopted. | | | | | | |
| ‡ | Increased family planning (74% in women age 20 and older, 33% in women younger than age 20) with increased safe abortion (100%) is more effective and less costly than current practice in Mexico. | | | | | | |
| § | Increased family planning (74% in women age 20 and older, 33% in women younger than age 20) with increased safe abortion (100%) and enhanced IpC/EmOC (100%/90%) has a cost-effectiveness ratio ranging from $40 per DALY averted (low-marginality states) to $390 per DALY averted (high-marginality states) compared to the next best nondominated strategy of increased family planning (33%/74%) with increased safe abortion (100%). | | | | | | |

**C. SUPPLEMENTAL DETAILS ABOUT PRODUCTIVITY LOSSES DUE TO MATERNAL MORTALITY:**

C.1 Losses due to Premature Mortality

Loss of future income due to premature mortality was calculated using the following method. First, we simulated a cohort of 100,000 women under current conditions in Mexico. We grouped the deaths in 5-year age bands. The total number of deaths in each group from all maternal conditions was multiplied by the average lost productivity for that age group (assuming an even distribution of women within the 5-year bands). To test whether this assumption had an impact, we also calculated the total lost productivity by single-year ages at time of predicted death. The lost productivity stream was calculated using data obtained from the International Labour Organization [47]. Using the percentage of women in each sector of the labor market, the actual annual salary for a woman working in that sector, and the assumption that 40% of women work in the informal sector in Mexico, valued as equal to the salary of the lowest formal sector worker, we calculated the average salary for a woman in Mexico at 31,595.56 in 2004 Mexican pesos. Following the methods outlined by Max et al [48], we calculated the lost productivity a woman would experience due to premature death from maternal causes. Briefly, while earnings were discounted annually at 3%, salaries were also increased annually by 1% to reflect earning increases that would occur over time. Using this weighted average salary per woman and calculating the lost future earning stream assuming each woman would have worked until age 65, an average woman who dies before the age of 20 would be expected to lose $83,036 (2001 U.S. $) in wages over the course of her lifetime. We produced Appendix Table 5, which indicates a woman’s lost future earning stream in both local currency and U.S. dollars, assuming she worked until age 65. By applying these earning losses to the simulated population, the approximate total lost productivity due to premature maternal mortality for a cohort of 100,000 women was estimated at nearly U.S. $11,000,000. Even with an upgrade to the coverage levels recommended in the Mother Baby Package, the losses due to premature maternal mortality are estimated at close to U.S. $6,000,000 per 100,000 women. For our most effective strategy, combining enhanced access to emergency obstetric care, increased safe abortion and increased family planning, the productivity losses are nearly U.S. $3,000,000 per 100,000 women.

**Appendix Table 5.** **Future productivity loss due to premature death***

| Age Death Occurs | Discounted future productivity lost due to early death  (2004 Mexican pesos) | Discounted future productivity lost due to early death  (2001 U.S. $) |
| --- | --- | --- |
| 20 or earlier | 966,927.86 | 83,036.03 |
| 21 | 935,332.30 | 80,322.72 |
| 22 | 904,350.25 | 77,662.10 |
| 23 | 873,969.78 | 75,053.15 |
| 24 | 844,179.23 | 72,494.85 |
| 25 | 814,967.14 | 69,986.23 |
| 26 | 786,322.27 | 67,526.32 |
| 27 | 758,233.62 | 65,114.17 |
| 28 | 730,690.37 | 62,748.87 |
| 29 | 703,681.95 | 60,429.49 |
| 30 | 677,197.96 | 58,155.14 |
| 31 | 651,228.22 | 55,924.96 |
| 32 | 625,762.75 | 53,738.09 |
| 33 | 600,791.75 | 51,593.67 |
| 34 | 576,305.63 | 49,490.90 |
| 35 | 552,294.97 | 47,428.96 |
| 36 | 528,750.53 | 45,407.05 |
| 37 | 505,663.27 | 43,424.41 |
| 38 | 483,024.30 | 41,480.26 |
| 39 | 460,824.92 | 39,573.86 |
| 40 | 439,056.60 | 37,704.48 |
| 41 | 417,710.97 | 35,871.40 |
| 42 | 396,779.81 | 34,073.92 |
| 43 | 376,255.09 | 32,311.33 |
| 44 | 356,128.90 | 30,582.97 |
| 45 | 336,393.51 | 28,888.17 |
| 46 | 317,041.34 | 27,226.28 |
| 47 | 298,064.93 | 25,596.66 |
| 48 | 279,457.00 | 23,998.69 |
| 49 | 261,210.39 | 22,431.74 |
| 50 | 243,318.08 | 20,895.22 |
| 51 | 225,773.19 | 19,388.53 |
| 52 | 208,568.99 | 17,911.10 |
| 53 | 191,698.84 | 16,462.36 |
| 54 | 175,156.27 | 15,041.74 |
| 55 | 158,934.91 | 13,648.72 |
| 56 | 143,028.54 | 12,282.74 |
| 57 | 127,431.02 | 10,943.28 |
| 58 | 112,136.37 | 9,629.84 |
| 59 | 97,138.70 | 8,341.90 |
| 60 | 82,432.25 | 7,078.96 |
| 61 | 68,011.36 | 5,840.55 |
| 62 | 53,870.49 | 4,626.19 |
| 63 | 40,004.20 | 3,435.41 |
| 64 | 26,407.15 | 2,267.74 |
| 65 | 13,074.13 | 1,122.76 |

*Productivity losses include only direct income while working and are based on average age of retirement of 65.

**D. SUPPLEMENTAL INFORMATION ABOUT PREVIOUS COST-EFFECTIVENESS ANALYSES FOR MATERNAL MORTALITY AND MORBIDITY:**

A comprehensive review of previously conducted cost-effectiveness analyses is being prepared as a separate manuscript but the following table is provided as an overview of prior economic evaluations of maternal health interventions.

Appendix Table 6: Overview of selected previous cost-effectiveness analyses for maternal mortality and morbidity

|  | **World Bank Report 1993 [49]** | **Maine et al 1991 [50]** | **Jha et al 1998 [51])** | **Prata et al 2004 [52]** | **Adam et al 2005 [18]** |
| --- | --- | --- | --- | --- | --- |
| **Model type** | unknown | non-model | non-model | Monte Carlo | Popmod |
| **Perspective** | not stated | not stated (3^rd^ party payor) | not stated (3^rd^ party payor) | not stated (3^rd^ party payor) | not stated (3^rd^ party payor) |
| **Target Population** | women of reproductive age and newborns | women of reproductive age | women of reproductive age and newborns | women of reproductive age and newborns | women of reproductive age and newborns |
| **Country** | developing countries | hypothetical developing country with population of 1 million | Guinea | 38 countries of sub-Saharan Africa | 14 epidemiological regions |
| **Time Horizon** | not stated | not stated | not stated | not stated | lifetime |
| **Health Outcomes** | Disability-Adjusted Life Years Saved | Number of Maternal Deaths averted | Life Years Saved, Discounted Life Years Saved | Number of Deaths Averted, and Maximal Number of Deaths Averted for a Fixed Budget | Yearly Disability-Adjusted Life Years Saved |
| **Main Outcome** | Average Incremental Cost-Effectiveness Ratio: Cost per Disability-Adjusted Life Year Saved | Average Incremental Cost-Effectiveness Ratio: Cost per Maternal Death Averted | Average Incremental Cost-Effectiveness Ratio: Cost per Life Year Saved | Average Incremental Cost-Effectiveness Ratio: Cost per Death Averted | Incremental Cost-Effectiveness Ratio: Cost per Disability-Adjusted Life Year Saved (Yearly) |

| Appendix Table 6: Overview of selected previous cost-effectiveness analyses for maternal mortality and morbidity (cont.)   |  | **World Bank Report 1993 [49]** | **Maine et al 1991 [50]** | **Jha et al 1998 [51])** | **Prata et al 2004 [52]** | **Adam et al 2005 [18]** | | --- | --- | --- | --- | --- | --- | | | | | | |
| --- | --- | --- | --- | --- | --- |
| **Relevant Interventions** | Prenatal and Delivery Care | Delivery using traditional birth attendants | Family Planning (Outreach clinics) | Prenatal Care | Prenatal Care (includes Tetanus immunization, screening and treatment of pre-eclampsia, bacteruria, syphilis) |
|  |  | Delivery using birth attendants trained to treat some causes of postpartum bleeding | Prenatal and Delivery Care (Centre-based) | Family Planning and Safe Abortion |  |
|  |  | Family planning services | Prenatal and Delivery Care (Hospital-based) | Safe Delivery (Normal Delivery +Management of Obstructed Labor) | Normal delivery by a skilled attendant |
|  |  | Prenatal care | Cesarean Section for Obstructed Labor | Eclampsia | Active management of the third stage of labor |
|  |  | 10 health centers | Family Planning, Prenatal/ Delivery Care (Centre-based) | Hemorrhage | Management of PPH |
|  |  | 5 health centers and 5 rural hospitals |  | Sepsis | Neonatal interventions* |
|  |  |  |  | Other MBP interventions | Treatment of pre-eclampsia/ eclampsia |
|  |  |  |  |  | Management of obstructed labor |
|  |  |  |  |  | Management of sepsis |
|  |  |  |  |  |  |

| Appendix Table 6: Overview of selected previous cost-effectiveness analyses for maternal mortality and morbidity (cont.)   |  | **World Bank Report 1993 [49]** | **Maine et al 1991 [50]** | **Jha et al 1998 [51])** | **Prata et al 2004 [52]** | **Adam et al 2005 [18]** | | --- | --- | --- | --- | --- | --- | | | | | | |
| --- | --- | --- | --- | --- | --- | --- | --- | --- | --- | --- | --- |
| **Type of costs included** | not fully described | equipment and supplies | Personnel, drugs, supplies, equipment, overhead | Personnel, drugs, supplies, equipment, overhead | Personnel, drugs, supplies, equipment, overhead, programmatic |
| **Sources of cost data** | costs are based on actual conditions | published studies and authors’ estimates | Primary data | Mother Baby Package Costing Spreadsheet and published studies | econometric estimation of country-specific hospital costs and program costs |
| **Year of $** | 1990 | not stated | 1994 | Not explicitly stated | 2000 |
| **Source (s) of effectiveness data** | Disease Control Priorities in Developing Countries (1993) and expert opinion | not explicitly stated | Disease Control Priorities in Developing Countries (1993) and expert opinion | WHO estimates from MBP. Iron supplement and malaria prevention (published studies) | for individual interventions based on published studies; for combination strategies assume a multiplicative effect |
| **Discount Rate** | not discounted | not discounted | 3%, applied to only benefits | not discounted | 3% |
| **Sensitivity Analyses** | No | No | No | Yes | Yes |
| *Neonatal care interventions included resuscitation, steroids for pre-term births, management of neonatal sepsis, asphyxia and jaundice, support and management of low birth weight, and community based case management for neonatal pneumonia. | | | | | |

**E. ADDITIONAL RESULTS AND SELECTED SENSITIVITY ANALYSES:**

E.1 Base Case Results

## Results from the base case as reported in the main manuscript (Table 2).

| **Appendix Table 7: Benefits, costs, and cost-effectiveness of current practice in Mexico (compared with no maternal care), and upgrading to the coverage rates in the WHO Mother Baby Package standard of care*** | | | | | | | | | |
| --- | --- | --- | --- | --- | --- | --- | --- | --- | --- |
| Strategy | | Mortality  (# deaths per 100,000) | Morbidity  (# events per 100,000) | Additional reduction in mortality vs. natural history, % | Additional reduction in morbidity vs. natural history, % | Costs, $  (average discounted lifetime) | Life expectancy  (average, discounted) | ICER ($/LY) | ICER ($/DALY) |
| Natural History | | 1,556 | 10,262 | --- | --- | 237.16 | 28.4010 | --- | --- |
| Current Practice in Mexico | | 175 | 4,149 | 88.7 | 59.6 | 502.87 | 28.6321 | † | † |
| MBP Standard of Care | | 92 | 2,755 | 94.1 | 73.2 | 371.82 | 28.6463 | 550 | 390 |
| * | LY = Life years, DALY = Disability adjusted life years, ICER = incremental cost-effectiveness ratio; MBP = Mother Baby Package. | | | | | | | | |
| † | *Current practice in Mexico* is dominated by the *MBP standard of care* since *MBP standard of care* is less costly and more effective. | | | | | | | | |

Results from the base case analysis (Table 3) with the additional strategy of current practice plus safe abortion.

| **Appendix Table 8: Maternal outcomes and cost-effectiveness of alternative strategies to improve maternal health compared with current practice in Mexico*** | | | | | | | |
| --- | --- | --- | --- | --- | --- | --- | --- |
| Strategy | | Mortality  (# deaths per 100,000) | Morbidity  (# events per 100,000) | Costs, $  (average discounted lifetime) | Life expectancy  (average discounted) | ICER ($/DALY) | Cost savings relative to current practice (per 100,000 women)† |
| *Current practice in Mexico* | | *175* | *4,149* | *502.87* | *28.6321* | *---* | *---* |
| Current Practice plus **increased FP** (33%/74%), **safe abortion** (100%) | | 101 (43%) | 2,261 (46%) | 386.23 | 28.6446 | ‡ | $ 11,600,000 |
| Current Practice plus **increased FP** (33%/74%), **safe abortion** (100%) and **enhanced IpC/EmOC** (100%/90%) | | 43 (75%) | 2,204 (47%) | 390.21 | 28.6555 | 300**§** | $ 11,200,000 |
| Current Practice plus **increased FP** (33%/74%), **enhanced IpC/EmOC** (100%/90%) | | 62 (64%) | 2,769 (33%) | 391.30 | 28.6519 | || | $ 11,100,000 |
| Current Practice plus **increased FP** (33%/74%) | | 119 (32%) | 2,825 (32%) | 397.30 | 28.6410 | || | $ 10,500,000 |
| Current Practice plus **safe abortion** (100%) and **enhanced IpC/EmOC** (100%/90%) | | 64 (64%) | 3,241 (22%) | 493.78 | 28.6522 | || | $ 900,000 |
| Current Practice plus **enhanced IpC/EmOC** (100%/90%) | | 92 (48%) | 4,068 (2%) | 495.03 | 28.6472 | || | $ 800,000 |
| Current Practice plus **safe abortion** (100%) | | 148 (16%) | 3,323 (20%) | 525.37 | 28.6370 | ** | ** |
| * | DALY = Disability adjusted life years, ICER = incremental cost-effectiveness ratio; IpC = intrapartum care; EmOC = emergency obstetric care; FP = family planning. Strategies increase coverage of specific interventions above the coverage rates in current practice. These include enhanced high-quality intrapartum care for all pregnant women (81% to 100%) and enhancing access to comprehensive emergency obstetric care for at least 90% of women (81% to 90%), safe abortion (from 50% to 100%), and FP (from 59% to 74% in women age 20 and older, and from 18% to 33% in women younger than age 20). All strategies are compared to current coverage; incremental cost-effectiveness ratios are assessed by ranking the strategies from the least costly to most costly and calculating the incremental change in costs and benefits with the next best strategy. For strategies that include enhanced IpC/EmOC access we assumed an incremental cost of $18.50 per woman requiring referral. | | | | | | |
| † | Cost savings relative to current practice (per 100,000 women) is an indicator of the resources that would be saved over the lifetime of a cohort of 100,000 women relative to current practice in Mexico if a particular strategy was adopted. This savings is calculated as the difference in total lifetime costs for a strategy compared to current practice, multiplied by 100,000. | | | | | | |
| ‡ | Increased family planning (74% in women age 20 and older, 33% in women younger than age 20) with increased safe abortion (100%) is more effective and less costly than current practice in Mexico. | | | | | | |
| **§** | Increased family planning (74% in women age 20 and older, 33% in women younger than age 20) with increased safe abortion (100%) and enhanced IpC/EmOC (100%/90%) has a cost-effectiveness ratio of $300/DALY compared to the next best strategy of increased family planning with increased safe abortion alone. | | | | | | |
| || | Strategy is less effective and more costly than increased family planning (74% in women age 20 and older, 33% in women younger than age 20) with increased safe abortion (100%) and enhanced IpC/EmOC (100%/90%) and is therefore formally dominated. Compared to current practice, these strategies are still cost saving. | | | | | | |
| ** | Strategy is less effective and more costly than increased family planning, safe abortion and enhanced IpC/EmOC access and is therefore formally dominated. | | | | | | |

E.2 Results of Sensitivity Analyses

We conducted one-way sensitivity analyses for all model parameters listed in Appendix Table 1. Overall cost-effectiveness results were robust over the plausible range of values for the risk of miscarriage and incomplete abortion; the incidence of severe anemia, severe preeclampsia/ eclampsia, obstructed labor, postpartum hemorrhage, and sepsis; the risk of mortality from severe preeclampsia/eclampsia, obstructed labor, postpartum hemorrhage, and sepsis; the relative risk of death due to severe anemia; the risk of morbidity from maternal complications (individually and globally); the probability of terminating a pregnancy with abortion or the relative proportion of safe versus unsafe abortions; the risk of morbidity from unsafe abortion; the effectiveness of severe preeclampsia/eclampsia, obstructed labor, postpartum hemorrhage, and sepsis treatment (either individually or globally); the failure rate associated with modern contraceptives (i.e., pill, condom, injectables, intrauterine device, sterilization); the cost associated with medical treatment of maternal disability (overall and for individual types of disability).

Cost-effectiveness results were most sensitive to assumptions about the overall cost of upgrading to the coverage levels and standard of care recommended by the Mother Baby Package, overall baseline effective coverage levels of maternal health interventions under the current standard of care in Mexico, and coverage level of family planning under current standard of care in Mexico

The percent reduction in mortality relative to the current standard of care in Mexico was most influenced by variations in assumptions about the overall baseline effective coverage of maternal health interventions and hospital-level interventions under current practice in Mexico; coverage of family planning alone under current practice; mortality risk of unsafe abortion, and the effectiveness of hospital-level maternal health interventions. However, variations in estimates for risks of mortality and intervention effectiveness did not impact cost and cost-effectiveness results owing to the limited impact of mortality alone on average total lifetime costs.

Results for selected sensitivity analyses are presented in the following sections.

E.3 Sensitivity Analysis of Overall Baseline Effective Coverage Rates

Within the states of Mexico, there is considerable heterogeneity with respect to the coverage of maternal health interventions [19]. For this sensitivity analysis, we lowered the levels of coverage for interventions considered to be current practice in Mexico by 25-50%. Total mortality and the magnitude of the reduction in maternal mortality was most sensitive to assumptions about the baseline effective coverage rates of interventions, and increased as the baseline effective coverage rates decreased. When the baseline effective coverage rate was decreased, the cost savings and cost-effectiveness ratios of most interventions became more favorable.

## **Appendix Table 9. Clinical effects and cost-effectiveness ratios for maternal health interventions with baseline intervention coverage levels reduced by 25% and 50%.***

| Strategy | | Mortality  (# deaths per 100,000) | | | Morbidity  (# events per 100,000) | | Costs  (average discounted lifetime) | | Life expectancy  (average discounted) | ICER ($/DALY) | | Cost savings relative to current practice (per 100,000 women)† |
| --- | --- | --- | --- | --- | --- | --- | --- | --- | --- | --- | --- | --- |
| **Overall intervention coverage levels reduced by 25%** | | | | | | | | | | | | |
| Current practice in Mexico | | | 382 | | 6,074 | | $ 518.86 | | 28.5968 | --- | | --- |
| Current Practice plus **increased FP** (33%/74%), **safe abortion** (100%) | | | 169 (56%) | | 2,355 (61%) | | $ 328.24 | | 28.6315 | ‡ | | $ 19,062,000 |
| Current Practice plus **increased FP** (33%/74%) | | | 192 (50%) | | 3,060 (50%) | | $ 342.20 | | 28.6271 | || | | $ 17,667,000 |
| Current Practice plus **increased FP** (33%/74%), **safe abortion** (100%) and **enhanced IpC/EmOC** (100%/90%) | | | 45 (88%) | | 2,238 (63%) | | $ 346.09 | | 28.6552 | 630 § | | $ 17,278,000 |
| Current Practice plus **increased FP** (33%/74%), **enhanced IpC/EmOC** (100%/90%) | | | 69 (82%) | | 2,944 (52%) | | $ 347.56 | | 28.6507 | || | | $ 17,130,000 |
| Current Practice plus **safe abortion** (100%) and **enhanced IpC/EmOC** (100%/90%) | | | 90 (76%) | | 4,460 (27%) | | $ 528.66 | | 28.6480 | ** | | ** |
| Current Practice plus **enhanced IpC/EmOC (100%/90%)** | | | 136 (64%) | | 5,850 (4%) | | $ 530.53 | | 28.6398 | ** | | ** |
| Current Practice plus **safe abortion** (100%) | | | 337 (50%) | | 4,688 (43%) | | $ 543.61 | | 28.6048 | ** | | ** |
| **Appendix Table 9. Clinical effects and cost (cont.)** | | | | | | | | | | | | |
| | Strategy | Mortality  (# deaths per 100,000) | Morbidity  (# events per 100,000) | Costs  (average discounted lifetime) | Life expectancy  (average discounted) | ICER ($/DALY) | Cost savings relative to current practice (per 100,000 women)† | | --- | --- | --- | --- | --- | --- | --- | | | | | | | | | | | | | |
| **Overall intervention coverage levels reduced by 50%** | | | | | | | | | | | | |
| Current practice Mexico | | | | 675 | | 8,204 | | $ 484.13 | 28.5474 | --- | --- | |
| Current Practice plus i**ncreased FP** (33%/74%), **safe abortion** (100%) | | | | 244 (64%) | | 2,450 (70%) | | $ 270.29 | 28.6173 | ‡ | $ 21,384,000 | |
| Current Practice plus **increased FP** (33%/74%) | | | | 271 (60%) | | 3,294 (60%) | | $ 287.18 | 28.6120 | || | $ 19,695,000 | |
| Current Practice plus **increased FP** (33%/74%), **safe abortion** (100%) and **enhanced IpC/EmOC** (100%/90%) | | | | 47 (93%) | | 2,273 (72%) | | $ 301.97 | 28.6548 | 710 § | $ 18,217,000 | |
| Current Practice plus **increased FP** (33%/74%), **enhanced IpC/EmOC** (100%/90%) | | | | 75 (89%) | | 3,119 (62%) | | $ 303.86 | 28.6494 | || | $ 18,027,000 | |
| Current Practice plus **safe abortion** (100%) | | | | 610 (10%) | | 6,133 (25%) | | $ 508.34 | 28.5589 | ** | ** | |
| Current Practice plus **safe abortion** (100%) and **enhanced IpC/EmOC** (100%/90%) | | | | 118 (83%) | | 5,704 (30%) | | $ 522.82 | 28.6436 | ** | ** | |
| Current Practice plus **enhanced IpC/EmOC** (100%/90%) | | | | 187 (72%) | | 7,787 (5%) | | $ 525.59 | 28.6315 | ** | ** | |
| **Appendix Table 9. Clinical effects and cost (cont.)** | | | | | | | | | | | | |
| * | DALY = Disability adjusted life years, ICER = incremental cost-effectiveness ratio; IpC = intrapartum care; EmOC = emergency obstetric care; FP = family planning. Strategies increase coverage of specific interventions above the coverage rates in current practice. These include enhanced high-quality intrapartum care for all pregnant women (81% to 100%) and enhancing access to comprehensive emergency obstetric care for at least 90% of women (81% to 90%), safe abortion (from 50% to 100%), and FP (from 59% to 74% in women age 20 and older, and from 18% to 33% in women younger than age 20). All strategies are compared to current coverage; incremental cost-effectiveness ratios are assessed by ranking the strategies from the least costly to most costly and calculating the incremental change in costs and benefits with the next best strategy. For strategies that include enhanced IpC/EmOC access we assumed an incremental cost of $18.50 per woman requiring referral. | | | | | | | | | | | |
| † | Cost savings relative to current practice (per 100,000 women) is an indicator of the resources that would be saved over the lifetime of a cohort of 100,000 women relative to current practice in Mexico if a particular strategy was adopted. | | | | | | | | | | | |
| ‡ | Increased family planning and safe abortion is more effective and less costly than current practice in Mexico. | | | | | | | | | | | |
| § | Increased family planning, safe abortion and enhanced IpC/EmOC access has a cost-effectiveness ratio of $630/DALY (25% reduction in baseline coverage) to $710/DALY (50% reduction in baseline coverage) compared to the next best strategy of increased family planning and safe abortion. | | | | | | | | | | | |
| || | Strategy is dominated by increased family planning, safe abortion and enhanced IpC/EmOC access. Compared to current practice, these strategies are still cost saving | | | | | | | | | | | |
| ** | Strategy is less effective and more costly than increased family planning, safe abortion and enhanced IpC/EmOC access and is therefore formally dominated. | | | | | | | | | | | |

E.4 Sensitivity Analysis of Baseline Effective Coverage Rates of Family Planning

For this sensitivity analysis, we lowered the levels of *current practice* coverage of family planning by 25-50%. The magnitude of reduction in mortality and morbidity were greatly influenced by assumptions about the coverage of family planning, and increased (as did total mortality and morbidity) as the coverage of family planning was decreased. In addition, the cost-savings of strategies associated with increased family planning coverage with a 25% reduction in family planning coverage doubled and tripled with a 50% reduction. These results demonstrate the significant clinical benefits and cost savings to be gained by the provision of family planning. By reducing unwanted pregnancies and births, increasing the coverage of family planning can avert costs and complications related to abortion, pregnancy, labor and delivery.

## **Appendix Table 10. Clinical effects and cost-effectiveness ratios for maternal health interventions with *current practice* family planning coverage levels reduced by 25% and 50%.***

| Strategy | | Mortality  (# deaths per 100,000) | | Morbidity  (# events per 100,000) | Costs  (average discounted lifetime) | Life expectancy  (average discounted) | ICER ($/DALY) | Cost savings relative to current practice (per 100,000 women)† |
| --- | --- | --- | --- | --- | --- | --- | --- | --- |
| **Family planning coverage levels reduced by 25%** | | | | | | | | |
| Current Practice in Mexico | | | 237 | 5,615 | $ 622.04 | 28.6222 | --- | --- |
| Current Practice plus **increased FP** (33%/74%), **safe abortion** (100%) | | | 101 (57%) | 2,261 (60%) | $ 386.23 | 28.6446 | ‡ | $ 23,581,000 |
| Current Practice plus **increased FP** (33%/74%), **safe abortion** (100%) and **enhanced IpC/EmOC** (100%/90%) | | | 43 (82%) | 2,204 (61%) | $ 390.21 | 28.6555 | 300 § | $ 23,183,000 |
| Current Practice plus **increased FP** (33%/74%), **enhanced IpC/EmOC** (100%/90%) | | | 62 (74%) | 2,769 (51%) | $ 391.30 | 28.6519 | || | $ 23,074,000 |
| Current Practice plus **increased FP** (33%/74%) | | | 119 (50%) | 2,825 (50%) | $ 397.30 | 28.641 | || | $ 22,474,000 |
| Current Practice plus **safe abortion** (100%) and **enhanced IpC/EmOC** (100%/90%) | | | 87 (63%) | 4,392 (22%) | $ 611.07 | 28.6486 | || | $ 1,097,000 |
| Current practice plus **enhanced IpC/EmOC** (100%/90%) | | | 124 (48%) | 5,506 (2%) | $ 612.26 | 28.6421 | || | $ 978,000 |
| Current Practice plus **safe abortion** (100%) | | | 200 (18%) | 4,503 (20%) | $ 652.25 | 28.6287 | ** | ** |
| **Appendix Table 10. Clinical effects and cost-effectiveness ratios for maternal health interventions with *current practice* family planning coverage levels reduced by 25% and 50%.* (cont.)** | | | | | | | | |
| Strategy | | | Mortality  (# deaths per 100,000) | Morbidity  (# events per 100,000) | Costs  (average discounted lifetime) | Life expectancy  (average discounted) | ICER ($/DALY) | Cost savings relative to current practice (per 100,000 women)† |
| **Family planning coverage levels reduced by 50%** | | | | | | | | |
| Current Practice in Mexico | | | 298 | 7,065 | $ 740.02 | 28.6125 | --- | --- |
| Current Practice plus **increased FP** (33%/74%), **safe abortion** (100%) | | | 101 (66%) | 2,261 (68%) | $ 386.23 | 28.6446 | ‡ | $ 35,379,000 |
| Current Practice plus **increased FP** (33%/74%), **safe abortion** (100%) and **enhanced IpC/EmOC** (100%/90%) | | | 43 (65%) | 2,204 (69%) | $ 390.21 | 28.6555 | 300 § | $ 34,981,000 |
| Current Practice plus **increased FP** (33%/74%), **enhanced IpC/EmOC** (100%/90%) | | | 62 (79%) | 2,769 (61%) | $ 391.30 | 28.6519 | || | $ 34,872,000 |
| Current Practice plus **increased FP** (33%/74%) | | | 119 (67%) | 2,825 (67%) | $ 397.30 | 28.6410 | || | $ 34,272,000 |
| Current Practice plus **safe abortion** (100%) and **enhanced IpC/EmOC** (100%/90%) | | | 131 (63%) | 6,665 (22%) | $ 843.02 | 28.6415 | ** | ** |
| Current practice plus **enhanced IpC/EmOC** (100%/90%) | | | 187 (48%) | 8,336 (2%) | $ 843.36 | 28.6319 | ** | ** |
| Current Practice plus **safe abortion** (100%) | | | 304 (15%) | 6,830 (20%) | $ 902.97 | 28.6122 | ** | ** |
| **Appendix Table 10. Clinical effects and cost-effectiveness ratios for maternal health interventions with *current practice* family planning coverage levels reduced by 25% and 50%.* (cont.)** | | | | | | | | |
| * | DALY = Disability adjusted life years, ICER = incremental cost-effectiveness ratio; IpC = intrapartum care; EmOC = emergency obstetric care; FP = family planning. Strategies increase coverage of specific interventions above the coverage rates in current practice. These include enhanced high-quality intrapartum care for all pregnant women (81% to 100%) and enhancing access to comprehensive emergency obstetric care for at least 90% of women (81% to 90%), safe abortion (from 50% to 100%), and FP (from 59% to 74% in women age 20 and older, and from 18% to 33% in women younger than age 20). All strategies are compared to current coverage; incremental cost-effectiveness ratios are assessed by ranking the strategies from the least costly to most costly and calculating the incremental change in costs and benefits with the next best strategy. For strategies that include enhanced IpC/EmOC access we assumed an incremental cost of $18.50 per woman requiring referral. | | | | | | | |
| † | Cost savings relative to current practice (per 100,000 women) is an indicator of the resources that would be saved over the lifetime of a cohort of 100,000 women relative to current practice in Mexico if a particular strategy was adopted. | | | | | | | |
| ‡ | Increased family planning and safe abortion is more effective and less costly than current practice in Mexico. | | | | | | | |
| § | Increased family planning, safe abortion, and enhanced IpC/EmOC access has a cost-effectiveness ratio of $300/DALY compared to the next best strategy of increased family planning and safe abortion. | | | | | | | |
| || | Strategy is less effective and more costly than increased family planning, safe abortion, and enhanced IpC/EmOC access and is therefore formally dominated. Compared to current practice, these strategies are still cost saving. | | | | | | | |
| ** | Strategy is less effective and more costly than increased family planning, safe abortion, and enhanced IpC/EmOC access and is therefore formally dominated. | | | | | | | |

E.5 Sensitivity Analysis of Mortality Risk Related to Unsafe Abortion

In the developing world, the risk of mortality related to unsafe abortion varies widely from roughly 0.1% in Latin America to 0.8% in Africa [39,40].. In the base case, we assumed that 17% of all pregnancies end in abortion, and assumed 50% occurred under “unsafe” conditions. The attributable mortality secondary to an unsafe abortion, or the case fatality rate of unsafe abortion, was assumed to be 0.001. We conducted sensitivity analyses in which we varied the underlying assumptions about the rate of abortion, the proportion of unsafe to safe abortions, and the case fatality rate. The figures below (Appendix Figures 2A, 2B, and 2C) each show a one way sensitivity analysis in which we vary the case fatality rate of unsafe abortion (Appendix Figure 2A), the underlying rate of abortion (Appendix Figure 2B), and the proportion of unsafe abortion (Appendix Figure 2C). The expected reduction in overall maternal morbidity and mortality are shown for each of these scenarios.

If the underlying rate of abortion in a pregnant woman is increased by 1.5, the case fatality rate due to unsafe abortion is increased to 0.002 or greater, the proportion of unsafe abortion to safe abortion is increased by 12.5%, and/or if the rate of attributable morbidity and costs of that morbidity are more than 2 times higher than our basecase assumptions, the incremental cost-effectiveness ratio associated with provision of safe abortion is less than 10% of the GDP and in many cases less than 5% of the GDP (corresponding to cost-effectiveness ratios of $100 to $500 per YLS or DALY averted). Although these values imply a situation in Mexico that may not reflect all states or the risk in women of all ages, they provide insight into the magnitude of the benefit by provision of safe abortion and demonstrate the value of the public health dollar invested in this regard. Provision of safe abortion for all women electing to terminate a pregnancy is very cost-effective under the base case assumptions. If there are areas in Mexico where the situation is worse than presented in our base case, the cost-effectiveness of improvements in safe abortion access become even more attractive.

**Appendix Fig. 2A.**

**Appendix Fig. 2B.**

**Appendix Fig. 2C.**

E.6 Sensitivity Analysis of Baseline Overall Effectiveness of Hospital-Level Interventions defined as part of Intrapartum Care/EmOC

For this sensitivity analysis, we reduced the overall effectiveness of hospital-level interventions such as treatment of severe preeclampsia/eclampsia, obstructed labor, postpartum hemorrhage, and sepsis by 25%-50%. The magnitude of reduction in mortality associated with maternal health strategies decreased as the effectiveness of hospital-level interventions decreased. For the most effective strategy of current practice plus increased family planning, safe abortion and enhanced IpC/EmOC access, there was a 27% (25% reduction in intervention effectiveness) to 40% (50% reduction in intervention effectiveness) decrease in the magnitude of mortality reduction compared to the base case. Morbidity outcomes were minimally affected because we assumed hospital-based interventions had no effect (except for management of obstructed labor) on reducing morbidity.

## **Appendix Table 11. Clinical effects and cost-effectiveness ratios for maternal health interventions with overall effectiveness of hospital-level interventions reduced by 25% and 50%.***

| Strategy | | Mortality  (# deaths per 100,000) | | Morbidity  (# events per 100,000) | Costs  (average discounted lifetime) | | Life expectancy  (average discounted) | ICER ($/DALY) | | Cost savings relative to current practice (per 100,000 women)† |
| --- | --- | --- | --- | --- | --- | --- | --- | --- | --- | --- |
| **Overall effectiveness of hospital-level interventions reduced by 25%** | | | | | | | | | | |
| Current Practice | | 275 | | 4,236 | $ 509.03 | | 28.6139 | --- | --- | |
| Current Practice plus **increased FP** (33%/74%), **safe abortion** (100%) | | 169 (39%) | | 2,321 (45%) | $ 390.72 | | 28.6316 | ‡ | $ 11,831,000 | |
| Current Practice plus **increased FP** (33%/74%), **safe abortion** (100%) and **enhanced IpC/EmOC** (100%/90%) | | 124 (55%) | | 2,279 (46%) | $ 395.75 | | 28.6401 | 480 § | $ 11,328,000 | |
| Current Practice plus **increased FP** (33%/74%), **enhanced IpC/EmOC** (100%/90%) | | 143 (48%) | | 2,843 (33%) | $ 396.84 | | 28.6366 | || | $ 11,219,000 | |
| Current Practice plus **increased FP** (33%/74%) | | 188 (32%) | | 2,885 (32%) | $ 401.77 | | 28.628 | || | $ 10,726,000 | |
| Current Practice plus **safe abortion** (100%) and **enhanced IpC/EmOC** (100%/90%) | | 182 (34%) | | 3,349 (21%) | $ 501.42 | | 28.6308 | || | $ 761,000 | |
| Current Practice plus **enhanced IpC/EmOC** (100%/90%) | | 210 (24%) | | 4,175 (1%) | $ 502.65 | | 28.6258 | || | $ 638,000 | |
| Current Practice plus safe abortion (100%) | | 248 (10%) | | 3,411 (19%) | $ 531.53 | | 28.6188 | ** | ** | |
| **Appendix Table 11. Clinical effects and cost-effectiveness ratios for maternal health interventions with overall effectiveness of hospital-level interventions reduced by 25% and 50%.* (cont.)**  | Strategy | Mortality  (# deaths per 100,000) | Morbidity  (# events per 100,000) | Costs  (average discounted lifetime) | Life expectancy  (average discounted) | ICER ($/DALY) | Cost savings relative to current practice (per 100,000 women)† | | --- | --- | --- | --- | --- | --- | --- |   **Overall effectiveness of hospital-level interventions reduced by 50%** | | | | | | | | | | |
| Current Practice | | | 384 | 4,322 | | $ 515.16 | 28.5942 | --- | --- | |
| Current Practice plus **increased FP** (33%/74%), **safe abortion** (100%) | | | 244 (38%) | 2,381 (45%) | | $ 395.19 | 28.6174 | ‡ | $ 11,997,000 | |
| Current Practice plus **increased FP** (33%/74%), **safe abortion** (100%) and **enhanced IpC/EmOC** (100%/90%) | | | 212 (45%) | 2,353 (46%) | | $ 401.27 | 28.6233 | 850 § | $ 11,389,000 | |
| Current Practice plus **increased FP** (33%/74%), **enhanced IpC/EmOC** (100%/90%) | | | 231 (40%) | 2,917 (33%) | | $ 402.35 | 28.6198 | || | $ 11,281,000 | |
| Current Practice plus **increased FP** (33%/74%) | | | 262 (32%) | 2,945 (32%) | | $ 406.23 | 28.6138 | || | $ 10,893,000 | |
| Current Practice plus **safe abortion** (100%) and **enhanced IpC/EmOC** (100%/90%) | | | 312 (19%) | 3,458 (20%) | | $ 509.03 | 28.6074 | || | $ 613,000 | |
| Current Practice plus **enhanced IpC/EmOC** (100%/90%) | | | 339 (12%) | 4,282 (1%) | | $ 510.24 | 28.6025 | || | $ 492,000 | |
| Current Practice plus **safe abortion** (100%) | | | 358 (7%) | 3,499 (19%) | | $ 537.66 | 28.599 | ** | ** | |
| **Appendix Table 11. Clinical effects and cost-effectiveness ratios for maternal health interventions with overall effectiveness of hospital-level interventions reduced by 25% and 50%.* (cont.)** | | | | | | | | | | |
| * | DALY = Disability adjusted life years, ICER = incremental cost-effectiveness ratio; IpC = intrapartum care; EmOC = emergency obstetric care; FP = family planning. Strategies increase coverage of specific interventions above the coverage rates in current practice. These include enhanced high-quality intrapartum care for all pregnant women (81% to 100%) and enhancing access to comprehensive emergency obstetric care for at least 90% of women (81% to 90%), safe abortion (from 50% to 100%), and FP (from 59% to 74% in women age 20 and older, and from 18% to 33% in women younger than age 20). All strategies are compared to current coverage; incremental cost-effectiveness ratios are assessed by ranking the strategies from the least costly to most costly and calculating the incremental change in costs and benefits with the next best strategy. For strategies that include enhanced IpC/EmOC access we assumed an incremental cost of $18.50 per woman requiring referral. | | | | | | | | | |
| † | Cost savings relative to current practice (per 100,000 women) is an indicator of the resources that would be saved over the lifetime of a cohort of 100,000 women relative to current practice in Mexico if a particular strategy was adopted. | | | | | | | | | |
| ‡ | Increased family planning with increased safe abortion is more effective and less costly than current practice in Mexico. | | | | | | | | | |
| § | Increased family planning with increased safe abortion and enhanced IpC/EmOC access has a cost-effectiveness ratio of $480/DALY (25% reduction in intervention effectiveness) to $850/DALY (50% reduction in intervention effectiveness) compared to the next best strategy of increased family planning with increased safe abortion alone. | | | | | | | | | |
| || | Strategy is dominated by family planning with increased safe abortion and enhanced IpC/EmOC access. Compared to current practice, these strategies are still cost saving | | | | | | | | | |
| ** | Strategy is less effective and more costly than increased family planning with increased safe abortion and enhanced IpC/EmOC access and is therefore formally dominated. | | | | | | | | | |

**REFERENCES**

1. Population Reference Bureau (2002) Family Planning Worldwide 2002 Data Sheet. Washington, DC: Population Reference Bureau.
2. Murray CJ and Lopez AD, editors (1998) Health Dimensions of Sex and Reproduction. Cambridge, MA: Harvard School of Public Health.
3. Cahuana-Hurtado L, Sosa-Rubi S, Bertozzi S (2004) The Application of the Mother Baby Package Reproductive Health Costing Spreadsheet in Morelos: National Institute of Public Health, Division of Health Economics and Policy, Mexico.
4. Lau CY, Qureshi AK (2002) Azithromycin versus doxycycline for genital chlamydial infections: a meta-analysis of randomized clinical trials. Sex Transm Dis 29: 497-502.
5. Teisala K, Heinonen PK, Aine R, Punnonen R, Paavonen J (1987) Second laparoscopy after treatment of acute pelvic inflammatory disease. Obstet Gynecol 69: 343-6.
6. Wølner-Hanssen P, Weström L (1983) Second-look laparoscopy after acute salpingitis. Obstet Gynecol 61: 702-4.
7. Brihmer C, Kallings I, Nord CE, Brundin J (1989) Second look laparoscopy; evaluation of two different antibiotic regimens after treatment of acute salpingitis. Eur J Obstet Gynecol Reprod Biol 30: 263-74
8. Magpie Trial Collaborative Group (2002) Do women with pre-eclampsia and their babies benefit from magnesium sulphate? The Magpie Trial: a randomised placebo controlled trial. Lancet 359: 1877-90.
9. Duley L, Gülmezoglu A, Henderson-Smart D (2003) Magnesium sulphate and other anticonvulsants for women with pre-eclampsia. Cochrane Database Syst Rev 2:CD000025.
10. [Duley L, Henderson-Smart D](http://www.ncbi.nlm.nih.gov.ezp1.harvard.edu/sites/entrez?Db=pubmed&Cmd=ShowDetailView&TermToSearch=10796146&ordinalpos=4&itool=EntrezSystem2.PEntrez.Pubmed.Pubmed_ResultsPanel.Pubmed_RVDocSum) (2000) Magnesium sulphate versus phenytoin for eclampsia. Cochrane Database Syst Rev 2:CD000128.
11. Johanson RB, Menon BK (2000) Vacuum extraction versus forceps for assisted vaginal delivery. Cochrane Database Syst Rev 2:CD000224.
12. Hofmeyr GJ, Kulier R (2000) External cephalic version for breech presentation at term. Cochrane Database Syst Rev 2: CD000083.
13. Schuitemaker N, van Roosmalen J, Dekker G, Van Dongen P, van Geijn H, Gravenhorst JB (1997) Maternal mortality after cesarean section in The Netherlands. Acta Obstetricia et Gynecologica Scandinavica 76: 332-4.
14. Hofmeyr GJ, Hannah ME (2003) Planned Caesarean section for term breech delivery. Cochrane Database Syst Rev 3:CD000166.
15. Gulmezoglu AM, Villar J, Ngoc NT, Piaggio G, Carroli G, et al (2001) WHO multicentre randomised trial of misoprostol in the management of the third stage of labour. Lancet 358: 689-95.
16. French LM, Smaill FM (2004) Antibiotic regimens for endometritis after delivery. Cochrane Database Syst Rev 4:CD001067.
17. French LM (2003) Prevention and treatment of postpartum endometritis. Curr Womens Health Rep 4: 274-9.
18. Adam T, Lim SS, Mehta S, Bhutta ZA, Fogstad H, et al (2005) Cost effectiveness analysis of strategies for maternal and neonatal health in developing countries. BMJ 331: 1107.
19. [Lozano R, Soliz P, Gakidou E, Abbott-Klafter J, Feehan DM, et al](http://www.ncbi.nlm.nih.gov/entrez/query.fcgi?db=pubmed&cmd=Retrieve&dopt=AbstractPlus&list_uids=17098091&query_hl=1&itool=pubmed_docsum) (2006) Benchmarking of performance of Mexican states with effective coverage. Lancet 368: 1729-41.
20. (1993) Maternal and child health. Maternal mortality in the Americas. Wkly Epidemiol Rec 68: 305-10.
21. Population Reference Bureau (2005) 2005 Women of Our World. Washington, DC. Population Reference Bureau.
22. Cahuana-Hurtado L, Sosa-Rubi S, Bertozzi S (2004) Cost of mother-child care in Morelos State. Salud Publica Mex 46: 316-25.
23. World Health Organization (1999) Mother–baby package costing spreadsheet user guide. Geneva: World Health Organization.
24. Engenderhealth (2003) Obstetric fistula needs assessment report: Findings from nine African countries. New York: Engenderhealth and UNFPA.
25. Ehrnborg C, Hakkaart-Van Roijen L, Jonsson B, Rutten FF, Bengtsson BA, Rosen T (2000) Cost of illness in adult patients with hypopituitarism. Pharmacoeconomics 17: 621-8.
26. Kent KC, Kuntz KM, Patel MR, Kim D, Klufas RA, et al (1995) Perioperative imaging strategies for carotid endarterectomy. An analysis of morbidity and cost-effectiveness in symptomatic patients. JAMA 274: 888-93.
27. Amowitz LL, Reis C, Iacopino V (2002) Maternal mortality in Herat Province, Afghanistan, in 2002: an indicator of women's human rights. JAMA 288: 1284-91.
28. Bartlett LA, Mawji S, Whitehead S, Crouse C, Dalil S, et al (2005) Where giving birth is a forecast of death: maternal mortality in four districts of Afghanistan, 1999-2002. Lancet 365: 864-70.
29. Harlap S, Shiono PH, Ramcharan S (1980) A life table of spontaneous abortions and the effects of age, parity and other variables. In: Porter IH, Hook EB, editors. Human Embryonic and Fetal Death. New York: Academic Press.
30. Menken J, Rahman MO (2006) Chapter 3: Reproductive Health. In: Merson MH, Black RE, Mills AJ, editors. International Public Health: Diseases, Programs, Systems, and Policies, 2nd ed. Sudsbury, MA: Jones and Bartlett Publishers: pp. 71-125.
31. Stamm WE, Guinan ME, Johnson C, Starcher T, Holmes KK, McCormack WM (1984) Effect of treatment regimens for Neisseria gonorrhoeae on simultaneous infection with Chlamydia trachomatis. N Engl J Med 310: 545-9.
32. Scholes D, Stergachis A, Heidrich FE, Andrilla H, Holmes KK, Stamm WE (1996) Prevention of pelvic inflammatory disease by screening for cervical chlamydial infection. N Engl J Med 334: 1362-6.
33. van Valkengoed IG, Morre SA, van den Brule AJ, Meijer CJ, Bouter LM, Boeke AJ (2004) Overestimation of complication rates in evaluations of Chlamydia trachomatis screening programmes--implications for cost effectiveness analyses. Int J Epidemiol 33: 416-25.
34. Paavonen J, Kiviat N, Brunham RC, Stevens CE, Kuo CC, et al (1985) Prevalence and manifestations of endometritis among women with cervicitis. Am J Obstet Gynecol 152: 280-6.
35. Brabin BJ, Hakimi M, Pelletier D (2001) An analysis of anemia and pregnancy-related maternal mortality. J Nutr 131(2 Suppl 2): 604S-614S.
36. Cleland J and Ali M (2003) Dynamics of contraceptive use. In: UN. Levels and Trends of Contraceptive Use as Assessed in 2002. New York: United Nations.
37. Trussell J, Kost K (1990) Contraceptive failure in the United States: an update. Stud Fam Plann 21: 51–54.
38. Alan Guttmatcher Institute (1996) An Overview of Clandestine Abortion in Latin America. New York: Alan Guttmacher Institute. 4 p.
39. Alan Guttmatcher Institute (1999). Facts in Brief: Induced Abortion Worldwide. New York: Alan Guttmacher Institute. pp. 1-2.
40. World Health Organization (2004) Unsafe abortion. Global and regional estimates of the incidence of unsafe abortion and associated mortality in 2000. Geneva: World Health Organization.
41. Elam-Evans LD, Strauss LT, Herndon J, Parker WY, Bowens SV, et al (2003) Abortion surveillance--United States, 2000. MMWR Surveill Summ 52: 1-32.
42. Alan Guttmacher Institute (2006) Preventing unsafe abortion and its consequences: Priorities for research and action. New York: Alan Guttmacher Institute.
43. Tomaro JB (1979). Abortion trend and practice in 6 states in northern Mexico. Estud Poblac 4: 61-84.
44. Ojha K, Philips Z, Darne FJ (2003) Diagnosing infertility in a district general hospital: a case-note and cost analysis. Hum Fertil 6: 169-73.
45. Hu D, Hook EW, Goldie SJ (2004) Screening for Chlamydia trachomatis in women 15 to 29 years of age: a cost-effectiveness analysis. Ann Intern Med 141: 501-13.
46. Avila JL, Fuentes, C, Tuiran R (2002) Indice de Marginacion a nivel localidad, 2000. Consejo Nacional de Población, Angel Urraza 1137, Mexico DF.
47. International Labour Organization. Available at [http://www.ilo.org](http://www.ilo.org/). Accessed 2007 Jul 13.
48. Max W, Rice DP, Sung HY, Michel M (2000) Valuing Human Life, Estimating the Present Value of Lifetime Earnings. Available at <http://repositories.cdlib.org/ctcre/esarm/PVLE2000>. Accessed 2007 Jul 13.
49. World Bank (1993) World Development Report 1993: Investing in Health, Washington, DC: World Bank.
50. Maine D (1991) Safe Motherhood Programs: Options and Issues. New York, NY: School of Public Health, Columbia University.
51. Jha P, Bangoura O, Ranson K (1998) The cost-effectiveness of forty health interventions in Guinea. Health Policy Plan 13: 249-62.
52. Prata N, Greig F, Potts M. Setting Priorities for Safe Motherhood Interventions in Resource Scarce Settings. Paper prepared for the 2004 Population Association of America Meeting, Boston, MA, April 1-2, 2004.
